# Modeling the Interactions Between Sodium Channels Provides Insight Into the Negative Dominance of Certain Channel Mutations

**DOI:** 10.3389/fphys.2020.589386

**Published:** 2020-11-05

**Authors:** Echrak Hichri, Zoja Selimi, Jan P. Kucera

**Affiliations:** Department of Physiology, University of Bern, Bern, Switzerland

**Keywords:** cardiac electrophysiology, sodium channels, sodium current, allosteric interactions, computer modeling, Markov models, statistical mechanics

## Abstract

**Background:**

Na_v_1.5 cardiac Na^+^ channel mutations can cause arrhythmogenic syndromes. Some of these mutations exert a dominant negative effect on wild-type channels. Recent studies showed that Na^+^ channels can dimerize, allowing coupled gating. This leads to the hypothesis that allosteric interactions between Na^+^ channels modulate their function and that these interactions may contribute to the negative dominance of certain mutations.

**Methods:**

To investigate how allosteric interactions affect microscopic and macroscopic channel function, we developed a modeling paradigm in which Markovian models of two channels are combined. Allosteric interactions are incorporated by modifying the free energies of the composite states and/or barriers between states.

**Results:**

Simulations using two generic 2-state models (C-O, closed-open) revealed that increasing the free energy of the composite states CO/OC leads to coupled gating. Simulations using two 3-state models (closed-open-inactivated) revealed that coupled closings must also involve interactions between further composite states. Using two 6-state cardiac Na^+^ channel models, we replicated previous experimental results mainly by increasing the energies of the CO/OC states and lowering the energy barriers between the CO/OC and the CO/OO states. The channel model was then modified to simulate a negative dominant mutation (Na_v_1.5 p.L325R). Simulations of homodimers and heterodimers in the presence and absence of interactions showed that the interactions with the variant channel impair the opening of the wild-type channel and thus contribute to negative dominance.

**Conclusion:**

Our new modeling framework recapitulates qualitatively previous experimental observations and helps identifying possible interaction mechanisms between ion channels.

## Introduction

Voltage-gated ion channels form the biophysical basis of action potential (AP) generation and propagation. Under physiological conditions, the sodium (Na^+^) current (I_Na_) carried by voltage-gated channels of the Na_v_1.X family ensure swift depolarization and rapid AP propagation in nerve axons, skeletal muscle, and cardiac muscle (Hodgkin, 1964; Hille, 2001; Barnett and Larkman, 2007; Matthews, 2013; Lieve and Wilde, 2015). In a voltage-dependent manner, Na^+^ channels change their conformation between permeable (open) and not permeable (e.g., closed, inactivated) states (Hille, 2001), ultimately leading to the upstroke of the AP. Because of this crucial role in AP generation, genetic variants of voltage-gated Na^+^ channels are frequently associated with pathologies of the central nervous system such as epilepsy (Wei et al., 2017; Nolan and Fink, 2018) or pain syndromes (Rühlmann et al., 2020), of skeletal muscle such as paramyotonia (Ke et al., 2017), and of the heart, where they can cause arrhythmias (Lieve and Wilde, 2015; Veerman et al., 2015).

In cardiomyocytes, Na_v_1.5 channels represent the principal Na^+^ channel isoform expressed. The pore-forming α-subunits of Na_v_1.5 channels are encoded by the *SCN5A* gene. Certain mutations of this gene are linked to life-threatening arrhythmias such as Brugada syndrome and long-QT syndrome type 3 (Lieve and Wilde, 2015; Veerman et al., 2015). Intriguingly, in cellular expression models, some *SCN5A* mutations negatively affect wild-type channel function leading to an effect called the dominant-negative (DN) effect. Although it was suggested that the DN effect is linked to a trafficking defect (Sottas and Abriel, 2016), it can occur even when both wild-type and variant channels are trafficked properly to the cell membrane (Clatot et al., 2018). Therefore, the detailed understanding of the function of cardiac Na^+^ channels is of high importance, not only for cardiac physiology but also for cardiology practice.

It was reported that Na_v_1.1, Na_v_1.2, and Na_v_1.5 channels form dimers, where their α-subunits interact physically with each other, leading to coupled channel gating (Clatot et al., 2017). To identify this interaction, biochemical and molecular biological approaches (crosslinking and photo-bleaching experiments) were combined with whole-cell electrophysiological recordings (binomial analysis based on Na^+^ current densities in cells transfected with different ratios of wild-type and DN variant genes), and single-channel recordings. The results indicate that two α-subunits can interact both directly and indirectly via the cytoplasmic protein 14-3-3. Mutating the 14-3-3 interaction sites and inhibiting 14-3-3 by difopein disrupted the molecular and biophysical interactions between two Na^+^ channel α-subunits (Clatot et al., 2017). More recently, evidence of dimerization and functional interaction was provided for Na_v_1.7 channels (Rühlmann et al., 2020). Altogether, these results strongly suggest that Na^+^ channels operate and gate as dimers. This notion challenges the conventional paradigm that these channels function as separate, individual, and non-interacting entities. To fully understand the consequences and implications of this paradigm shift, new analyses and models need to be developed in which the functional unit underlying the Na^+^ current is a Na^+^ channel dimer rather than a single channel.

Clatot et al. (2017) analyzed their single-channel recordings from channel pairs by counting the number of sweeps exhibiting, at predefined times after a voltage clamp activation step, 1 (level 1, L1) or 2 (level 2, L2) open channels. They showed that the L2 count is decreased and the L1 count is increased upon disrupting the interaction between the Na^+^ channels by difopein, indicating that the channels tend to be open together. In the present work, we analyze these L1 and L2 counts further using the χ^2^ test and Fisher’s exact test to establish the significance of this observation. We also quantify the interaction using Shannon’s entropy, a measure from information theory.

Next, we designed models of ion channel function incorporating interactions between two channels with the aim to recapitulate the findings of Clatot et al. (2017). The first biophysical model for the gating of Na^+^ and K^+^ currents was proposed by Hodgkin and Huxley (1952) and their formalism is still used in many cardiac cell models (Courtemanche et al., 1998; ten Tusscher et al., 2004; O’Hara et al., 2011). Markovian models however are more versatile (Colquhoun and Hawkes, 1995b; Hille, 2001; Bondarenko et al., 2004; Milescu et al., 2005; Fink and Noble, 2009; Perissinotti et al., 2018; Asfaw and Bondarenko, 2019), because they permit simulations of both stochastic single-channel behavior and macroscopic ensemble currents, and more precisely account for the binding of drugs to specific states (Rudy and Silva, 2006; Silva et al., 2009; Moreno et al., 2011).

Thus, we implemented a framework combining two Markovian ion channel models. Allosteric interactions between channels are then introduced in agreement with principles of statistical mechanics by changing the free energies of composite states and of the energy barriers between composite states. This approach allows simulating and describing the effect of the interactions on both the microscopic (stochastic single-channel gating) and the macroscopic (ensemble average) behaviors of the Na^+^ current. We conducted simulations and sensitivity analyses for a 2-state (closed-open), 3-state (closed-open-inactivated), and a full cardiac sodium channel model (Clancy and Rudy, 1999, a 6-state channel model). The sensitivity analyses pinpointed that an increased free energy of composite states consisting of one closed and one open channel is a key factor leading to coupled gating.

We furthermore modeled the consequences of the p.L325R variant of Na_v_1.5, a variant which was reported in a patient with Brugada syndrome and which is known to exert a DN effect (Keller et al., 2005; Clatot et al., 2017). When we incorporated the biophysical properties of the p.L325R variant into our channel pair framework, our model showed that the DN variant negatively affected the biophysical function of the wild-type channel through the allosteric interaction. This highlights the notion that DN effects can arise directly from molecular interactions between Na^+^ channels.

## Materials and Methods

### Quantification of the Interaction Between Two Channels Under Non-stationary Conditions

Various methods have been developed to demonstrate or quantify the interactions between two or more ion channels based on recordings at the single-channel level (Yeo et al., 1989; Fredkin and Rice, 1991; Chung and Kennedy, 1996). These approaches however presuppose that the system of channels is at equilibrium and its behavior is stationary. These assumptions clearly do not pertain to the Na^+^ current upon an activation protocol because the ensemble average current changes with time.

Clatot et al. (2017, 2018) analyzed their recordings of voltage-gated Na^+^ channel pairs by counting the number of sweeps containing 1 and 2 open channels (called Level 1 (L1) and Level 2 (L2), respectively) as a function of time and reported the time course of L1 and L2. Note that L0 (zero open channels) corresponds to *n*–L1–L2, *n* being the number of sweeps. In the following, we elaborate on further analyses that can be conducted on such L0, L1 and L2 counts.

We consider f_0_, f_1_, and f_2_, the fractions of sweeps with 0, 1, and 2 open channels at a given time during the sweeps, calculated as L0/*n*, L1/*n*, and L2/*n*, with f_0_ + f_1_ + f_2_ = 1. These fractions represent finite sample approximations of the true probabilities of observing 0, 1 or 2 open channels, and converge to the true probabilities p_0_, p_1_, and p_2_ as *n* is increased. Thus, at a given time *t*, each triplet {L0(*t*), L1(*t*), L2(*t*)} forms a sample (i.e., a finite sample approximation) from a discrete 3-element distribution with expectation values *n*⋅p_0_(*t*), *n*⋅p_1_(*t*) and n⋅p_2_(*t*).

For two non-interacting (independent) channels labeled A and B, f_0_, f_1_, and f_2_ can be described as follows (for large *n* and in the limit as *n* goes to infinity):

$${\text{f}}_{0}={\text{f}}_{\text{A},\text{shut}}\cdot {\text{f}}_{\text{B},\text{shut}}$$

$${\text{f}}_{1}={\text{f}}_{\text{A},\text{open}}\cdot {\text{f}}_{\text{B},\text{shut}}+{\text{f}}_{\text{A},\text{shut}}\cdot {\text{f}}_{\text{B},\text{open}}$$

(1) $${\text{f}}_{2}={\text{f}}_{\text{A},\text{open}}\cdot {\text{f}}_{\text{B},\text{open}}$$

where f_A,shut_, f_A,open_, f_B,shut_, and f_B,open_ represent the fractions of sweeps with channel A, respectively B, shut (non-conducting, i.e., closed or inactivated), respectively open at a given time.

If the two channels are identical and non-interacting, then f_A,shut_ = f_B,shut_ = f_shut_ and f_A,open_ = f_B,open_ = f_open_ (with f_open_ + f_shut_ = 1), and the following is expected:

$${\text{f}}_{0}={\text{f}}_{\text{shut}}^{2}$$

$${\text{f}}_{1}=2\cdot {\text{f}}_{\text{open}}\cdot {\text{f}}_{\text{shut}}$$

(2) $${\text{f}}_{2}={\text{f}}_{\text{open}}^{2}$$

For interacting channels, Eqs. 1 and 2 do not necessarily hold and f_0_, f_1_, and f_2_ must be described in a more general manner as

$${\text{f}}_{0}={\text{f}}_{\text{A},\text{shut};\text{B},\text{shut}}$$

$${\text{f}}_{1}={\text{f}}_{\text{A},\text{open};\text{B},\text{shut}}+{\text{f}}_{\text{A},\text{shut};\text{B},\text{open}}$$

(3) $${\text{f}}_{2}={\text{f}}_{\text{A},\text{open};\text{B},\text{open}}$$

If the two channels are identical (interacting or non-interacting), and if only one channel is open, it is impossible to distinguish in a patch clamp experiment which of the channels is open. Thus, if the channels are identical and indistinguishable, the probability that it is either A or B open is 0.5:

(4) $${}_{P\left(A\mathrm{open}|\mathrm{one}\mathrm{channel}\mathrm{open}\right)=P\left(B\mathrm{open}|\mathrm{one}\mathrm{channel}\mathrm{open}\right)=1/2.}$$

We underline that this equal probability of A or B being open is not only valid for two identical non-interacting channels, but also for two identical interacting channels, as long as the interaction is symmetric (the action of A on B is the same as the action of B on A).

Thus, for identical channels (interacting or not),

(5) $${\text{f}}_{\text{A},\text{open};\text{B},\text{shut}}={\text{f}}_{\text{A},\text{shut};\text{B},\text{open}}$$

From Eqs. 3 and 5, f_*s*__hut_ and f_open_, the fraction of sweeps in which a given channel is shut, respectively open (at a given time), can be estimated individually for each identical indistinguishable channel as

$${\text{f}}_{\text{A},\text{shut}}={\text{f}}_{\text{A},\text{shut};\text{B},\text{shut}}+{\text{f}}_{\text{A},\text{shut};\text{B},\text{open}}={\text{f}}_{0}+{\text{f}}_{1}/2$$

$${\text{f}}_{\text{B},\text{shut}}={\text{f}}_{\text{A},\text{shut};\text{B},\text{shut}}+{\text{f}}_{\text{A},\text{open};\text{B},\text{shut}}={\text{f}}_{0}+{\text{f}}_{1}/2$$

$${\text{f}}_{\text{A},\text{open}}={\text{f}}_{\text{A},\text{open};\text{B},\text{shut}}+{\text{f}}_{\text{A},\text{open};\text{B},\text{open}}={\text{f}}_{1}/2+{\text{f}}_{2}$$

(6) $${\text{f}}_{\text{B},\text{open}}={\text{f}}_{\text{A},\text{shut};\text{B},\text{open}}+{\text{f}}_{\text{A},\text{open};\text{B},\text{open}}={\text{f}}_{1}/2+{\text{f}}_{2}$$

with f_A,shut_ = f_B,shut_ = f_shut_ and f_A,open_ = f_B,open_ = f_open_.

Therefore, given a triplet of observed f_0_, f_1_, and f_2_, f_open_ and f_shut_ can be estimated for identical indistinguishable channels as

$${\text{f}}_{\text{shut}}={\text{f}}_{0}+{\text{f}}_{1}/2$$

(7) $${\text{f}}_{\text{open}}={\text{f}}_{1}/2+{\text{f}}_{2}$$

Note that the same result is obtained for non-interacting channels from Eq. 2. Thus, Eq. 7 pertains to any pair of identical indistinguishable channels irrespective of whether the channels interact or not.

In a next step, we estimate from f_open_ and f_shut_ (calculated in Eq. 7) the fractions $\bar{f_{0}}$, $\bar{{\text{f}}_{1}}$, and $\bar{{\text{f}}_{2}}$ that would be expected in the absence of any interaction (the overbar denotes the expected fraction). For this purpose, we use Eq. 1 (underlining that f_open_ and f_shut_ are now the values calculated from f_0_, f_1_, and f_2_ using Eq. 7). Thus, we define

$$\bar{{\text{f}}_{0}}={\text{f}}_{\text{shut}}^{2}={\left({\text{f}}_{0}+{\text{f}}_{1}/2\right)}^{2}$$

$$\bar{{\text{f}}_{1}}=2\cdot {\text{f}}_{\text{open}}\cdot {\text{f}}_{\text{shut}}=2\cdot \left({\text{f}}_{1}/2+{\text{f}}_{2}\right)\cdot \left({\text{f}}_{0}+{\text{f}}_{1}/2\right)$$

(8) $$\bar{{\text{f}}_{2}}={\text{f}}_{\text{open}}^{2}={\left({\text{f}}_{1}/2+{\text{f}}_{2}\right)}^{2}$$

From these expected fractions, the $\bar{\text{L}0},\bar{\mathrm{L1}}$ and $\bar{\text{L}2}$ counts expected in the absence of interaction can then be calculated by multiplication with *n*. The significance of the difference between the observed distribution {f_0_, f_1_, f_2_} and the distribution {$\bar{f_{0}}$, $\bar{{\text{f}}_{1}}$, $\bar{{\text{f}}_{2}}$} calculated in Eq. 8 assuming the null hypothesis of independence (absence of interactions) can be ascertained using the χ^2^ test, a statistical method typically used to ascertain independence (Howell, 2011). Similarly, the significance of the difference between the observed distribution {L0, L1, L2} and the calculated distribution {$\bar{\text{L}0},\bar{\text{L}1}$, $\bar{\text{L}2}$} can be ascertained using Fisher’s exact test (Sprent, 2011).

In summary, the flow of the analysis is as follows. First, we divide the observed L0, L1 and L2 counts by *n* to calculate f_0_, f_1_, and f_2_. Then we calculate f_shut_ and f_open_ according to Eq. 7. Next, using f_shut_ and f_open_, we calculate $\bar{f_{0}}$, $\bar{{\text{f}}_{1}}$, and $\bar{{\text{f}}_{2}}$ according to Eq. 8, and $\bar{\text{L}0},\bar{\text{L}1}$ and $\bar{\text{L}2}$ are calculated from $\bar{f_{0}}$, $\bar{{\text{f}}_{1}}$, and $\bar{{\text{f}}_{2}}$ by multiplication with *n*. Finally, {L0, L1, L2} and {$\bar{\text{L}0},\bar{\text{L}1}$, $\bar{\text{L}2}$} are compared statistically. In the Supplementary Material, we illustrate and support our derivation of Eqs. 1–8 using contingency tables, elaborate on the suitability of the χ^2^ test and Fisher’s exact test, and provide a numerical example.

The difference between the distributions {f_0_, f_1_, f_2_} and {$\bar{f_{0}}$, $\bar{{\text{f}}_{1}}$, $\bar{{\text{f}}_{2}}$} can also be quantified using measures based on information theory, starting from Shannon’s entropy (Shannon, 1948). For indistinguishable interacting channels, Shannon’s entropy is obtained from the observed values of f_0_, f_1_, and f_2_ as

(9) $$\text{S}=-\left({\text{f}}_{0}\log\left({\text{f}}_{0}\right)+2\frac{{\text{f}}_{1}}{2}\log\left(\frac{{\text{f}}_{1}}{2}\right)+{\text{f}}_{2}\log\left({\text{f}}_{2}\right)\right),$$

and using the values $\bar{f_{0}}$, $\bar{{\text{f}}_{1}}$, and $\bar{{\text{f}}_{2}}$ calculated by assuming the absence of interaction, the entropy is

(10) $$\bar{\text{S}}=-\left(\bar{f_{0}}\log\left(\bar{f_{0}}\right)+2\frac{\bar{f_{1}}}{2}\log\left(\frac{\bar{f_{1}}}{2}\right)+\bar{f_{2}}\log\left(\bar{f_{2}}\right)\right).$$

The entropy difference

(11) $$\Delta \text{S}=\text{S}-\bar{\text{S}}$$

quantifies the information lost by assuming independence in the calculation given by Eqs. 7 and 8. If the channels are independent, ΔS is expected to be 0; otherwise, ΔS is expected to be negative, which will reflect the presence of a phenomenon that generates order (lower entropy) in the distribution of f_0_, f_1_, and f_2_.

The ensemble average current I_average_ can be reconstructed from f_open_ (see Eq. 7) as

(12) $${\text{I}}_{\text{average}}={\text{f}}_{1}{\text{i}}_{\text{ch}}+2{\text{f}}_{2}{\text{i}}_{\text{ch}}=2\cdot {\text{f}}_{\text{open}}\cdot {\text{i}}_{\text{ch}}$$

where i_ch_ is the single-channel current (assumed to be the same for the 2 channels). It can equivalently be reconstructed as

$${\text{I}}_{\text{average}}=\bar{f_{1}}{\text{i}}_{\text{ch}}+2\bar{f_{2}}{\text{i}}_{\text{ch}}=\left(2\cdot {\text{f}}_{\text{open}}\cdot {\text{f}}_{\text{shut}}+2\cdot {\text{f}}_{\text{open}}^{2}\right){\text{i}}_{\text{ch}}=$$

(13) $$2\cdot {\text{f}}_{\text{open}}\cdot \left({\text{f}}_{\text{shut}}+{\text{f}}_{\text{open}}\right){\text{i}}_{\text{ch}}=2\cdot {\text{f}}_{\text{open}}\cdot {\text{i}}_{\text{ch}},$$

since, by definition of $\bar{f_{0}}$, $\bar{{\text{f}}_{1}}$, and $\bar{{\text{f}}_{2}}$ (Eq. 8), f_0_, f_1_, f_2_ and $\bar{f_{0}}$, $\bar{{\text{f}}_{1}}$, $\bar{{\text{f}}_{2}}$ correspond to the same f_shut_ and f_open_. Thus, ΔS also quantifies the information lost when computing the ensemble average, because this computation disregards the interdependence of f_0_, f_1_, and f_2_.

Of note, the analyses presented in this section can be conducted separately at any time point during the voltage clamp protocol, even if the system is not at equilibrium. For these analyses to be valid, the only condition is that the system must be ergodic, that is, it must exhibit statistically the same behavior over repeated successive experiments (recording sweeps).

### Markovian Modeling of Channel Pairs

#### From Markovian Models of Single Channels to a Markovian Model of a Channel Pair

We start with the formulation of a single ion channel as a continuous-time discrete-state Markov model, a widely accepted approach in ion channel electrophysiology (Colquhoun and Hawkes, 1981; Qin et al., 2000; Milescu et al., 2005; Keener, 2009; Siekmann et al., 2012). Such a model consists of N possible conformational states (e.g., closed, open, inactivated) that can be graphically represented by a state diagram, that is, a directed graph in which labeled nodes represent the states and bidirectional arrows represent possible transitions. Mathematically, the probabilities of the individual states are described by an N-dimensional probability column vector **p**, whose elements sum up to 1, and a N × N matrix **Q** (which is voltage-dependent, and, if voltage changes, also time-dependent) representing the transition rate coefficients (hereafter referred to as transition rates, or simply as rates) of the possible transitions between the different states. The dynamics of the model are then described by the following master equation:

(14) $$\frac{\text{d}p}{\text{d}t}=\mathrm{Qp},$$

where the element q_ij_ in the i^th^ row and the j^th^ column of **Q** represents the transition rate from state j to state i (or 0 if this transition is not possible) and the diagonal elements of **Q** are constructed such that the sum of every column of **Q** is 0 to, which guarantees that the sum of **p** remains 1. Note that for the master equation, we adopted here the form favored in physical chemistry, which is also the form used by Keener (2009). Other work on Markovian ion channel modeling (e.g., Colquhoun and Hawkes, 1981; Qin et al., 2000; Milescu et al., 2005; Siekmann et al., 2012) use the transposed notation d**p**/d*t* = **pQ** in which **p** is a row vector and in which the rows of **Q** sum to 0. Both formulations are, however, equivalent because they are the transpose of each other.

**FIGURE 1:**
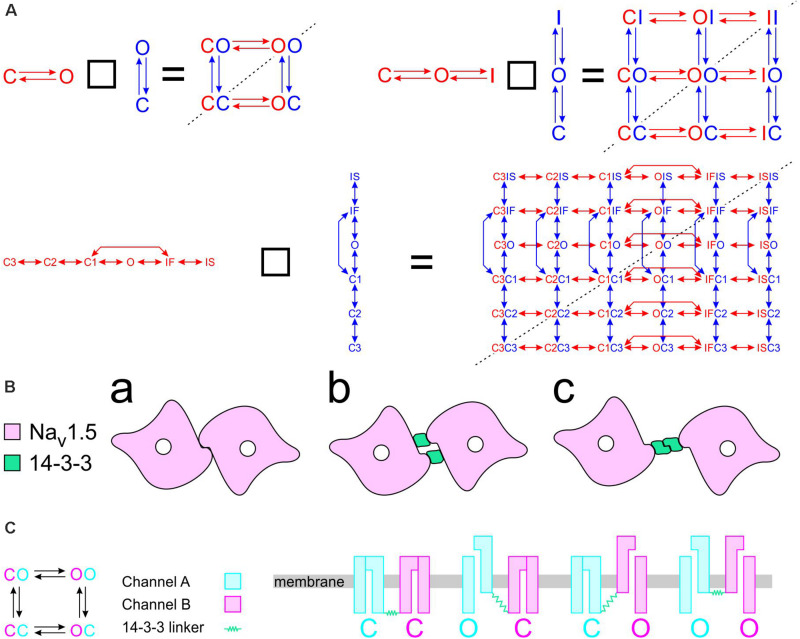
Modeling a pair of channels. **(A)** Composition of two Markovian channel models. *Top left:* Two generic 2-state models. *Top right:* Two generic 3-state models. *Bottom:* Two instances of the Na^+^ channel model of Clancy and Rudy (1999). The symbol “□” denotes the Cartesian graph product. Note the symmetries about the diagonals (dashed lines). **(B)** Cartoon of a channel dimer (viewed from an axis perpendicular to the membrane). a: direct contact; b: linked by two 14-3-3 proteins; c: linked by a 14-3-3 dimer. Because proteins are chiral, a symmetry is expected for the binding pattern and a rotation by 180° is expected to leave the entire structure unchanged. In this situation, identical channels are indistinguishable. **(C)** Cartoon illustrating how interaction between channels may change the free energy of certain combinations of states. In this example, opening of one channel stretches the 14-3-3 linker (represented as a green spring) and the potential energy accumulated in the stretched linker is added to the free energy of the composite CO and OC states.

To construct a compound model of two channels, we consider that any state of the first channel can be associated to any state of the second, assuming that no additional state is generated. We furthermore consider that any transition occurring within one channel can occur when the other channel is in any of its possible states. Graphically, such a composition can be represented by the Cartesian product of the corresponding graphs (Figure 1A) for two 2-state models (closed-open), two 3-state models (closed-open-inactivated), and two 6-state models of the cardiac Na^+^ channel model of Clancy and Rudy (1999). If the channels do not interact, the corresponding rates within each channel (i.e., columns of horizontal red arrows and rows of vertical blue arrows in Figure 1A) are equal to those in the original single-channel models. The two channels are thus paired into a single functional unit. Any two Markovian models can be combined in this manner, even if they have different numbers of states or different graphs.

Mathematically, the composition of two non-interacting channels A (with N_A_ states) and B (with N_B_ states), determined by rate matrices **Q**_A_ and **Q**_B_ and probability vectors **p**_A_ and **p**_B_, can be constructed as

(15) $${\text{Q}}_{\text{AB}}={\text{Q}}_{\text{A}}⊕{\text{Q}}_{\text{B}}={\text{Q}}_{\text{A}}⊗{\text{I}}_{\text{B}}+{\text{I}}_{\text{A}}⊗{\text{Q}}_{\text{B}}$$

where ⊕ denotes the Kronecker sum, ⊗ denotes the Kronecker tensor product, and **I**_A_ and **I**_B_ are the N_A_ × N_A_ and N_B_ × N_B_ identity matrices, respectively (Siekmann et al., 2016). The result, **Q**_AB_, is an N_A_N_B_ × N_A_N_B_ rate matrix describing the compound model as

(16) $$\frac{\text{d}{\text{p}}_{\text{AB}}}{\text{d}t}=Q_{\text{AB}}{\text{p}}_{\text{AB}}$$

with **p**_AB_ being the N_A_N_B_-dimensional probability vector of composite states. The two summands in Eq. 15 correspond to the transitions within one channel while the state of the other channel is fixed, in Figure 1A represented respectively by the red horizontal and blue vertical sets of arrows. In the Supplementary Material, we provide the definitions of the Kronecker product and sum and we write out the terms in Eqs. 15 and 16 for two 2-state C↔O Markovian models to illustrate the block structure of the matrices.

From **p**_AB_, the separate probability vectors **p**_A_ and **p**_B_ for each individual channel can be obtained by summing over corresponding elements of **p**_AB_ using matrix operators **O**_A_ and **O**_B_ (consisting of ones and zeros) as

(17) $${\text{p}}_{\text{A}}={\text{O}}_{\text{A}}{\text{p}}_{\text{AB}}\;\text{and}\;{\text{p}}_{\text{B}}={\text{O}}_{\text{B}}{\text{p}}_{\text{AB}}$$

with

(18) $$O_{\text{A}}={\text{I}}_{\text{A}}⊗1_{\text{B}}^{\text{T}}\;\text{and}\;{\text{O}}_{\text{B}}=1_{\text{A}}^{\text{T}}⊗{\text{I}}_{\text{B}}$$

where $1_{\text{A}}^{\text{T}}$ and $1_{\text{B}}^{\text{T}}$ are row vectors of ones (the superscript ^T^ denotes transposition) with N_A_ and N_B_ elements, respectively (not to be confused with the identity matrices **I**_A_ and **I**_B_). In the Supplementary Material, we provide an example of the construction of **O**_A_ and **O**_B_ for a composite model of two 2-state channels.

If the channels do not interact but are subject to the same experimental conditions, **p**_AB_ obeys the following relation (Siekmann et al., 2016):

(19) $${\text{p}}_{\text{AB}}={\text{p}}_{\text{A}}⊗{\text{p}}_{\text{B}}$$

This relation asserts the independence of both channels by stating that the probability of finding channel A in state i and channel B in state j is equal to the product of the probability of finding channel A in state i and the probability of finding channel B in state j, if both channels are considered separately.

For any model **Q**, the equilibrium (steady-state) probability vector **s** can be found by solving the system

(20) Qs=0

under the constraint that all elements of **s** sum up to 1. Without interaction, Eq. 19 is also valid for the equilibrium probability vector **s**_AB_ of **Q**_AB_:

(21) $${\text{s}}_{\text{AB}}={\text{s}}_{\text{A}}⊗{\text{s}}_{\text{B}},$$

where **s**_A_ and **s**_B_ are the steady-state probability vectors of **Q**_A_ and **Q**_B_, respectively.

#### Pair of Identical and Indistinguishable Channels

If the two channels are identical, then **Q**_A_ = **Q**_B_, **p**_A_ = **p**_B_, and a symmetry appears. This symmetry can be conceptualized graphically by the symmetry of the compound graphs in Figure 1A about their diagonal. The symmetry exchanges the colors of the arrows and symbols, but does not change the compound model. If, in addition, the dynamics of the two channels (or the channels themselves) cannot be distinguished by any available experimental procedure, they are *de facto* indistinguishable, and a physical, conceptual, or mathematical permutation of the channels will not change the paired system. Because proteins are chiral, a geometrical symmetry is likely to appear (Cintas, 2013), as illustrated in Figure 1B. Note that if we considered interacting objects that are themselves symmetric (e.g., upon reflection in a plane), further arrangements having a plane symmetry would also be possible. However, Na^+^ channels do not possess a plane of symmetry, and the only symmetric structure that can be built is one with a rotational symmetry of 180°.

Consequently, the probability of finding channel A in state i and channel B in state j is equal to the probability of finding channel A in state j and channel B in state i. From this, in the absence of any further information (i.e., if all possible open states have the same conductance and the only available experimental observation is that only one of the two channels is conductive), Eq. 4 can be deduced. Note that this is a consequence of A and B being identical and does not rely on chirality.

#### Incorporating Conservative Interactions (Preserving Microscopic Reversibility)

One important principle in Markovian modeling is the principle of microscopic reversibility, also known as detailed balance (Kelly, 1979; Hille, 2001). This principle states that at equilibrium, the flux of probability from state X to state Y (the product of the steady-state probability of X and the rate constant going from X to Y) is equal to the flux of probability from Y to X (Kelly, 1979; Hille, 2001). It can be formalized mathematically as

(22) QS=SQ,Ti.e.,QSissymmetric

where **S** is a diagonal matrix formed with the elements of the steady-state probability vector **s** (satisfying **Qs** = **0**). Equivalently, this principle can be formalized by Kolmogorov’s criterion (Kelly, 1979): for every loop in the Markovian model, the product of the transition rates in one direction along the loop must be equal to the product of the transition rates in the reverse direction; thus, there is no preferential motion in a given direction around a loop. From the viewpoint of statistical mechanics, this principle states that, in the long term, no energy is produced or consumed by the channel (conservation of energy).

Transition rates (i.e., rate coefficients) are usually described by Arrhenius’ and Eyring’s theories (Tsien and Noble, 1969; Jack et al., 1975; Hille, 2001; Sigg and Bezanilla, 2003; Sigg, 2014). The transition rate r_ij_ from state i to state j is related to the height of the energy barrier $\Delta G_{\text{ij}}^{‡}$ (considered at the level of the single molecule, not at the molar level) encountered when transiting from i to j as

(23) $${\text{r}}_{\text{ij}}=\kappa \frac{\text{kT}}{h}e^{-\Delta G_{\text{ij}}^{‡}/\text{kT}}$$

where k is Boltzmann’s constant, *h* is Planck’s constant, κ is a constant factor (transmission coefficient) and T is absolute temperature (we consider in this work a constant physiological temperature of 37°C = 310.15 K). The transition rate r_ij_then appears as element q_ji_ in the j^th^ row and the i^th^ column of **Q**. Taking the logarithm of Eq. 23 shows that the barrier height is related to the logarithm of the rate. Thus, Kolmogorov’s criterion can also be expressed as follows: the sum of the ascended energy barriers in one direction along the loop must be equal to the sum of the ascended energy barriers in the reverse direction, and no net energy is gained or lost after completing the loop.

By construction, the composition of models presented in Figure 1A and the mathematical formulation of non-interacting models in Eq. 15 preserve microscopic reversibility if the original models also do so (a proof outline is provided in the Supplementary Material).

Concerning allosteric interactions, energy may be exchanged between interacting units (e.g., between the α-subunits of Na^+^ channels or between the α-subunits and a linker protein), but in the long term, no net energy (e.g., metabolic, chemical) is generated or dissipated. Consequently, we consider that allosteric interactions must preserve microscopic reversibility. Using the energy landscape analogy, it can be understood that modifying the energy level of a state (represented by a trough in the energy landscape), the energy of a barrier (the altitude of a barrier), or any combination of such operations will preserve microscopic reversibility.

Raising the energy of a state by an amount E corresponds to scaling all rates of the transitions exiting that state by *e*^E/kT^. For *E* > 0 (the energy of the state is raised), the exiting transitions are accelerated. For example, for *E* = kT, these transitions are accelerated *e*-fold. For *E* < 0 (the energy of the state is lowered), the exiting transitions are slowed. Mathematically, this corresponds to multiplying the corresponding column of **Q** by *e*^E/kT^. If **Q** represents a composite model of two channels having each the same state diagram (e.g., Figure 1A), the energy of two corresponding state compositions S_a_S_b_ and S_b_S_a_ (e.g., CO and OC in Figures 1A,C) must be changed by the same amount (unless a = b). For this case, two columns of **Q** are multiplied by *e*^E/kT^. Figure 1C provides an example illustrating how the change in free energy of a composite state relates to potential energy accumulated in the interaction between the channels.

Conversely, raising the energy of a barrier by an amount E corresponds to scaling the two transition rates between the two states separated by this barrier by *e*^–E/kT^. For *E* > 0, these transitions are slowed, and for *E* < 0, these transitions are accelerated. This corresponds to scaling the two corresponding entries of **Q** (four entries if **Q** describes a symmetric composite model) and recalculating the diagonal entries of **Q** such that the sum of each column of **Q** remains 0.

The operations of changing the energy of a state and changing the energy of a barrier preserve Kolmogorov’s criterion. Furthermore, these operations commute and can thus be applied on **Q** in any order. In the Supplementary Material, we provide a few examples of such operations and illustrate their commutativity. Any other change to **Q** that cannot be decomposed as a combination of these elementary operations violates microscopic reversibility.

### Deterministic and Stochastic Simulations of Ion Channel Function

#### Deterministic Simulations

We ran both deterministic and stochastic simulations of ion channel function in response to a voltage step applied at time *t* = 0, mimicking a voltage clamp step protocol. In deterministic simulations of single channels and of composite non-interacting or interacting channels, the vector of state probabilities was computed using matrix exponentials (Colquhoun and Hawkes, 1995a; Siekmann et al., 2012) as

(24) $$\text{p}\left(t\right)=e^{tQ}\text{p}\left(0\right),$$

where **p**(*t*) is the vector of state probabilities at time *t* and **p**(0) is the initial condition at time 0 (start of the voltage step). Eq. 24 is the analytical solution of Eqs. 14 and 16 when **Q** does not change with time (as during a voltage step protocol to a given potential). For existing Na^+^ channel models (e.g., Clancy and Rudy, 1999) subjected to a voltage clamp activation protocol, **Q** was computed as a function of the step potential and **p**(0) was computed as the steady-state solution **s** of Eq. 20 with **Q** as function of the holding potential. In simulations of compound channels (A and B, non-interacting or interacting), the separate probability vectors **p**_A_ and **p**_B_ for each individual channel were computed according to Eqs. 17 and 18. Macroscopic currents were reconstructed by extracting the elements of **p**_A_ and **p**_B_ corresponding to open states and multiplying them by the corresponding maximal conductance or maximal current. Similarly, the probabilities (as a function of time) to observe 0, 1, or 2 open channels (p_0_, p_1_, and p_2_) were obtained from **p**_A_ and **p**_B_. The probabilities p_0_, p_1_, and p_2_ represent the expectation values of the fractions f_0_, f_1_, and f_2_ (see section “Quantification of the interaction between two channels under non-stationary conditions”) when the number of sweeps tends toward infinity.

#### Stochastic Simulations

Stochastic simulations of transitions between different states were carried out as described previously (Milescu et al., 2005; Lemay et al., 2011). In brief, if the state of a model (single or compound channel) is known at time *t*, then the probability to find the model in any given state at time *t* + Δ*t* is determined by the transition probability matrix **A** given by

(25) $$\text{A}=e^{\text{Δ}t<cps:bf>Q</cps:bf>}$$

Unless specified otherwise, we used a time step Δ*t* of 0.001 ms. The element in the *i*^th^ column and *j*^th^ row of **A** represents the probability of the model to be in state *j* at time *t* + Δ*t* if the model is in state *i* at time *t*. Every column of **A** sums to 1. The state of the model at time *t* + Δ*t* was therefore simulated by drawing a state at random from the multinomial distribution given by the corresponding column of **A**. The stochastic behavior of the model during a predefined simulation time was then obtained by sequential iteration. Single-channel currents or currents from a channel pair were then computed by adding unitary currents through open states. The entire process was repeated *n* times to simulate *n* sweeps, from which ensemble average currents were computed. For every time step Δ*t*, the sweeps containing 0, 1, or 2 open channels were counted (L0, L1, and L2). From these counts, the probabilities f_0_, f_1_, and f_2_ were estimated by respectively dividing L0, L1, and L2 by *n*. As initial conditions, channels were set in the closed state for 2-state and 3-state models (see Figure 1A). For Na^+^ channel simulations, the initial state was obtained by drawing it randomly from the multinomial distribution given by the steady-state vector **s** at holding potential.

Simulations were validated by reducing Δ*t* 10 times. No difference in the results was observed, indicating that the Δ*t* of 0.001 ms was sufficiently small. To validate our framework, we also implemented Gillespie’s algorithm (Gillespie, 1977) and repeated selected simulations (detailed in the “Results” section).

### Quantitative Analyses

#### Quantification of the Synchrony of Gating

Clatot et al. (2017, 2018) also examined the proportion of simultaneous openings (or closings) by calculating the proportion of consecutive openings (or closings) separated by a time interval shorter than a predefined threshold value.

In our stochastic simulations, rather than using a threshold value, we examined histograms of the distribution of the time interval between consecutive openings of the two channels (without any other event in between and without regard to which channel opened first) and between two consecutive closings, respectively. The distributions were then summarized by their mean and median, as markers for subsequent analysis.

For stationary channel behavior, these distributions can be derived analytically for any given Markovian model (e.g., Yeo et al., 1989; Fredkin and Rice, 1991; Colquhoun and Hawkes, 1995b). However, for a non-stationary situation, to our knowledge, no analytical method exists to compute such distributions. In addition, these distributions may vary with time. Thus, the histograms were computed explicitly in the stochastic simulations over the entire duration of the voltage step.

#### Markers for Macroscopic/Ensemble Average Currents

We defined different markers to characterize the influence of interactions between channels on the kinetics of macroscopic or ensemble average currents (determined from deterministic simulations).

For the compound of two C↔O models (Figure 1A, left), we computed the steady-state level of the current, its maximal derivative, the peak of p_1_ and the timing of this peak, and the maximum of p_2_. For the compound of two C↔O↔I models (Figure 1A, right) and of two Clancy-Rudy models (Figure 1A, bottom), we computed the peak and the time of the peak current, the maximal and minimal derivative (during activation and inactivation), the time constant of fast inactivation, and the peaks of p_1_ and p_2_ with the timing of these peaks.

#### Sensitivity Analysis

The energy of individual states and barriers were systematically varied to determine the influence of these energy changes on the markers defined above, in a manner similar to that described by Sobie (2009). For each state or barrier, the energy was changed by an amount going from –2 kT to +2 kT in steps of 1 kT (kT corresponds at physiological temperature to 0.616 kcal/mol or 26.7 meV). The sensitivity of a given marker to the energy change was then quantified as the regression slope of the natural logarithm of the marker vs. the energy. The quality of the correlation was assessed using the square of the correlation coefficient *r*^2^. We note that this sensitivity analysis is local (in the sense that it starts from an already parametrized Markov model), hence exploring sensitivity around this particular point.

### Computational Aspects

All simulations and analyses were conducted in MATLAB (R2015b, The MathWorks, Natick, MA, United States). Unless specified otherwise, simulations were run for 3 ms with a constant time step Δ*t* of 0.001 ms. In stochastic simulations, *n* = 1000 sweeps were simulated for each model/interaction. Matrix exponentials and Kronecker products were computed using the functions “expm” and “kron.”

The MATLAB code is available on Zenodo ( doi: 10.5281/zenodo.4064027).

## Results

### Quantitative Analysis of the Interaction Between Na^+^ Channels in Published Experimental Data

**FIGURE 2:**
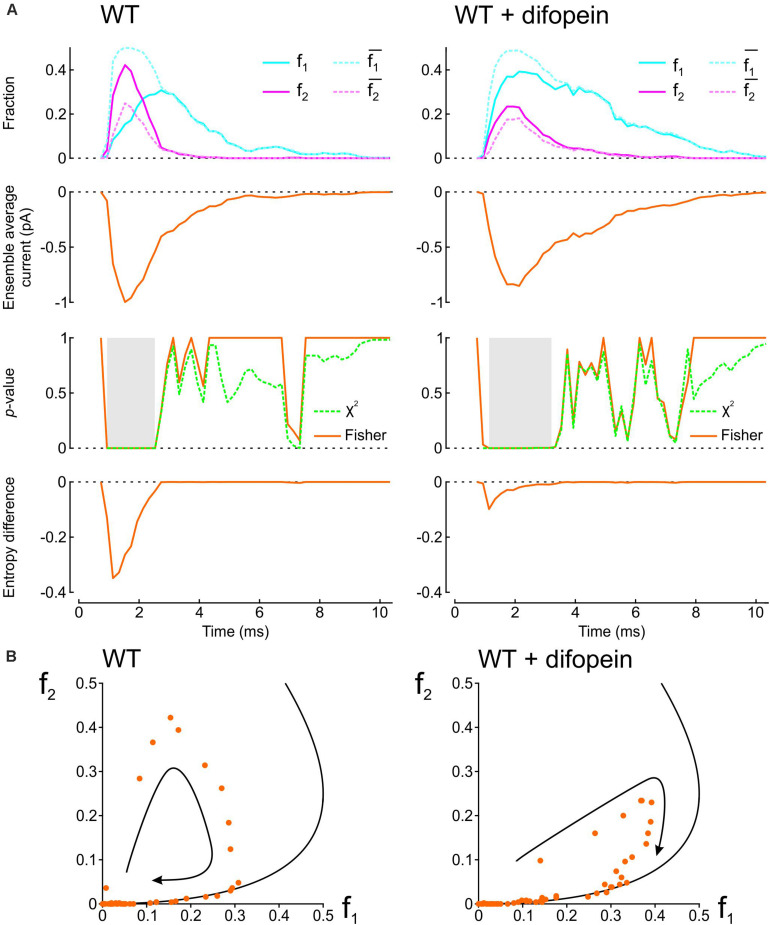
Analysis of single-channel data published by Clatot et al., 2017, (Supplementary Figure S8), licensed under a Creative Commons Attribution 4.0 International License (https://creativecommons.org/licenses/by/4.0/). In patch clamp recordings from wild-type cardiac Na^+^ channel pairs submitted to an activation step to −20 mV, Clatot et al. (2017) counted at every sampling time the number of sweeps with one open channel (L1) and two open channels (L2). L1 and L2 were extracted from the vectorized figure. Dividing L1 and L2 by the number of sweeps yields f_1_ and f_2_, the fractions of sweeps with one or two open channels at a given time. **(A)** Analysis of experimental data in the absence (*left*) vs. presence (*right*) of difopein. *Top row:* Raw fractions f_1_ and f_2_ (solid cyan and magenta lines) and fractions $\bar{{\text{f}}_{1}}$ and $\bar{{\text{f}}_{2}}$ that would be expected in the absence of interaction (Eqs. 7 and 8, dotted cyan and magenta lines). *Second row:* Ensemble average currents computed using Eqs. 12 and 13 (assuming a single-channel current i_ch_ of −1 pA). *Third row: p*-value (as a function of time) of the χ^2^ test (green) and Fisher’s exact test (orange) for independence. Intervals during which *p* < 0.01 are highlighted in gray. *Fourth row:* Entropy difference ΔS computed using Eqs. 9–11. **(B)** Plots of f_2_ vs. f_1_ (dots) for experiments without (*left*) and with (*right*) difopein. The curved arrows indicate the direction of the trajectories. The black curves represent the expected relationship in the absence of interactions ($\bar{{\text{f}}_{2}}$ vs. $\bar{{\text{f}}_{1}}$).

To demonstrate how our proposed analyses provide insight into the interaction between two Na^+^ channels, we applied them in Figure 2 to patch clamp data from pairs of human wild-type (WT) cardiac Na^+^ channels (Na_v_1.5) reported by Clatot et al. (2017). These experiments were conducted in the presence vs. absence of difopein, a protein believed to disrupt the interaction between the channels via 14-3-3. In their recordings, Clatot et al. (2017) counted at every sampling time the number of sweeps with one open channel (L1) and two open channels (L2). Dividing L1 and L2 by the number of sweeps *n* yields f_1_ and f_2_, the fractions of sweeps with one or two open channels (solid curves in the top panels of Figure 2A). From f_1_ and f_2_, we then computed $\bar{{\text{f}}_{1}}$ and $\bar{{\text{f}}_{2}}$, the expected fractions if the channels were independent (dotted curves in the top panels of Figure 2A). Reconstructed ensemble average currents are shown in the second row of panels in Figure 2A. From the onset of the average Na^+^ current and around the Na^+^ current peak, f_1_ was smaller than $\bar{{\text{f}}_{1}}$ and f_2_ was greater than $\bar{{\text{f}}_{2}}$, confirming the tendency of the channels to be open together rather than separately. Interestingly, later during inactivation, these differences suggestive of Na^+^ channel interaction disappeared (overlap of dotted and solid curves). With difopein, moreover, similar (although smaller) differences are apparent between f_1_ and $\bar{{\text{f}}_{1}}$ as well as between f_2_ and $\bar{{\text{f}}_{2}}$, which suggests that difopein did not fully disrupt the interaction between the two channels.

We then computed the statistical significance of the difference between observed L1 and L2 counts and counts expected for independent channels using the χ^2^ test and Fisher’s exact test. Both tests yielded *p*-values very close to 0 during the onset and peak of the average Na^+^ current without difopein (for some time points, *p* < 10^–20^), statistically confirming the interaction between the channels. The significant difference disappeared during inactivation after about 1 ms following the peak of the ensemble average current. With difopein, the significant interaction at the onset and peak of the average Na^+^ current was also present.

To quantify the interaction using information theory, we computed the entropy difference ΔS (Eqs. 9–11), shown in the fourth row of Figure 2A. The negative ΔS around the peak average Na^+^ current corroborates the interaction. Consistent with the other data shown in Figure 2A, ΔS returned to 0 during inactivation and ΔS was less negative in the presence of difopein.

Another approach to graphically reveal the interaction between the channels is to plot f_2_ vs. f_1_ (Figure 2B). During activation, f_2_ increased considerably faster than f_1_. After passing the peak average Na^+^ current, f_2_ rapidly decreased while f_1_ continued to increase. Importantly, the data points were initially located far from the curve corresponding to a binomial distribution for independent channels, but collapsed onto this curve later during inactivation. In the f_2_ vs. f_1_ phase space, the fractions thus followed a clockwise loop trajectory bounded below by the curve for independent channels. With difopein, the loop was still apparent, albeit with a reduced magnitude. Altogether, our analysis substantiates the interaction between Na^+^ channels, indicates that this interaction vanishes during inactivation, and shows it is only partially suppressed by difopein.

### Lessons From a Composite Pair of 2-State Markov Models

We first formulated a composite model consisting of the two simplest Markov channel models consisting each of one closed (C) and one open state (O). The channels (labeled A and B) are identical, and without interaction their opening rate is 1 ms^–1^ and their closing rate is 2 ms^–1^. We conducted deterministic and stochastic simulations after placing both channels in the C state as initial condition. Intuitively, the notion that Na^+^ channels tend to open and to close together suggests that composite CO and OC states must be relatively unstable with a decreased probability. This decreased stability could result from increased free energy of these composite states, as illustrated in Figure 1C. We therefore ran control simulations in the absence of interactions, and simulations in which the free energy of the composite CO and OC states was raised by 2 kT. Note that the CO and OC states can be distinguished in the simulations, but in a patch clamp experiment, such composite states would be indistinguishable.

**FIGURE 3:**
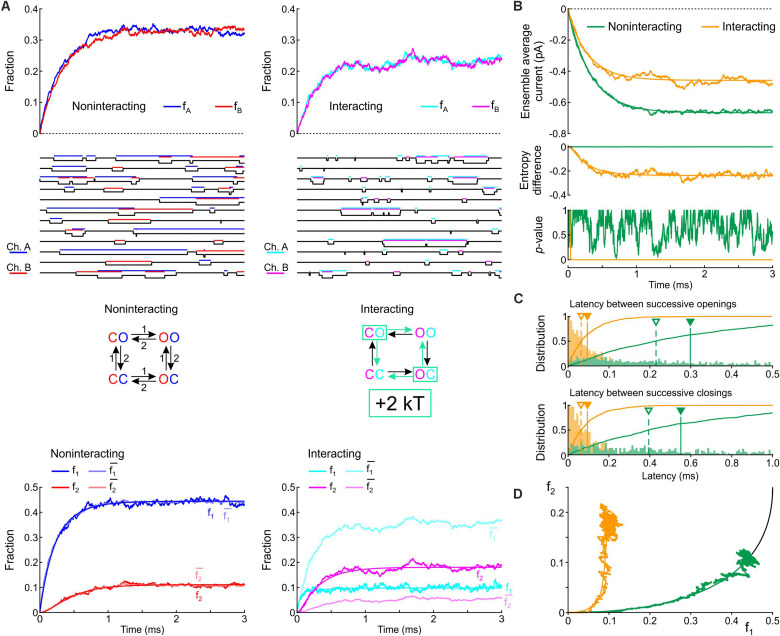
Simulated gating behavior of a pair of 2-state channels (C: closed ↔ O: open; opening rate: 1 ms^–1^; closing rate: 2 ms^–1^) in the absence of interaction and upon raising the energy of the composite CO and OC states by 2 kT. As initial condition (*t* = 0), the channels were all put into the C state. **(A)** *Top row:* Fractions f_A_ and f_B_ of the individual channels A and B being open in the absence (blue/red, *left*) and presence (cyan/magenta, *right*) of the interaction, reconstructed from *n* = 1000 simulated sweeps. *Second row:* Simulated sweeps. The simulated current is represented in black; the intervals during which the channels were open are marked by colored overbars. *Third row:* Corresponding graphs of the composite Markovian models of non-interacting and interacting channels (numbers correspond to rates in ms^–1^; colored arrows indicate the rates accelerated by the interaction). *Bottom row:* Fractions f_1_ and f_2_ of finding one or two channels open for non-interacting and interacting channels (color legend in the inset), and expected fractions without interaction (lighter hues) computed from Eqs. 7 and 8. Continuous lines were computed using deterministic simulations. **(B)** Ensemble average current (*top*), entropy difference (*middle*) and *p*-value of Fisher’s exact test (*bottom*) for the non-interacting (green) and interacting (orange) channel pair. The continuous lines were generated by deterministic simulations. **(C)** Histograms of the latency between successive openings (*top*) and closings (*bottom*), and cumulative histograms of this latency (solid curves) for the non-interacting (green) and interacting (orange) channel pair. Filled triangles and solid vertical lines indicate means; open triangles and dashed vertical lines indicate median values. **(D)** f_2_ vs. f_1_ plots without (green) and with channel interaction (orange). The continuous curves were obtained from deterministic simulations. The black curve is the theoretical expectation for independent channels.

Figure 3A shows corresponding state diagrams, individual realizations of stochastic simulations (sweeps), individual fractions f_A_ and f_B_ of the *n* = 1000 sweeps with channels A or B being open, and corresponding fractions f_1_ and f_2_. The bottom panels of Figure 3A also show the fractions $\bar{{\text{f}}_{1}}$ and $\bar{{\text{f}}_{2}}$ computed from f_1_ and f_2_ under the assumption that the channels are independent. For the non-interacting pair, the sweeps illustrate that individual channel openings and closings are uncorrelated. For the interacting pair, the gating behavior clearly differs: in the sweeps, channels visibly tend to be open together and consecutive openings and closings tend to occur in closer temporal proximity. Irrespective of the presence or absence of interaction, the fractions f_A_ and f_B_ (top panels of Figure 3A) evolve from 0 to their stationary equilibrium (reflecting activation) with f_A_ ≈ f_B_ because the channels are identical. For the interacting channels, the steady-state probability is lower (∼0.21) compared to the non-interacting channels (0.33). The plots of f_1_, f_2_, $\bar{{\text{f}}_{1}}$, and $\bar{{\text{f}}_{2}}$ (Figure 3A, *bottom*) show that, for the non-interacting pair, $\bar{{\text{f}}_{1}}$ remains close to f_1_ and $\bar{{\text{f}}_{2}}$ remains close to f_2_, as expected. Introducing the interaction increased f_2_ and decreased f_1_. Moreover, for the interacting pair, f_1_ < $\bar{{\text{f}}_{1}}$ and f_2_ > $\bar{{\text{f}}_{2}}$, consistent with the finding shown in Figure 2A during activation.

Figure 3B shows the average Na^+^ current, the entropy difference and the *p*-value (Fisher’s exact test) computed as in Figure 2A. Consistent with the observation that the interaction increases f_A_ and f_B_, the ensemble average current was about 50% larger (in absolute value) for the interacting pair. The entropy difference converged to a negative value near –0.21 for the interacting pair, while it remained 0 as expected for the non-interacting pair. The *p*-value immediately dropped near 0 for the interacting channels (refuting the null hypothesis of independence), while it fluctuated between 0 and 1 without interaction. Figure 3C shows histograms of the latency between successive openings and closings and cumulative histograms of these latencies. The interaction drastically skewed the histograms toward shorter latencies and decreased severalfold the mean and median latency between successive openings and successive closings. Lastly, the f_2_ vs. f_1_ plots (Figure 3D) show that the interaction shifted the trajectory upwards and leftwards from the theoretical expectation for independent channels, while the trajectory remained near the theoretical attractor in the f_1_-f_2_ phase space in the absence of interaction. These observations are consistent with the analysis conducted on experimental data (Figure 2A) during the activation phase.

In simulations in which the opening rate of the single-channel model was set to 2 ms^–1^ and its closing rate to 1 ms^–1^ (Supplementary Figure S1), the effects of channel interaction were qualitatively similar, with the exception that it increased, rather than decreased, the fractions f_A_ and f_B_ and the ensemble average current. Taken together, the simulations of a pair of 2-state channels suggest that an increased free energy of composite CO/OC states may be involved in the biophysical mechanism leading to coupled gating and the experimentally observed behaviors of f_1_ and f_2_.

**FIGURE 4:**
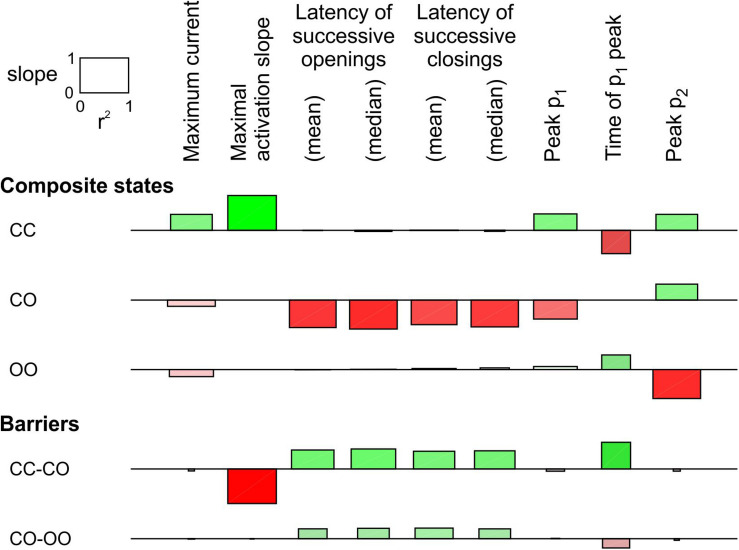
Sensitivity analysis for the pair of 2-state channels (C: closed ↔ O: open; opening rate: 2 ms^–1^; closing rate: 1 ms^–1^). The free energy of every composite state and barrier (labels on the left) was individually varied by an amount going from –2 kT to +2 kT. Then, the influence of this variation was quantified by the regression slope of the natural logarithm of observable parameters (labels on the top) and the *r*^2^ value of this regression (scale rectangle in the top left corner). Positive correlations are shown as green bars above the horizontal lines. Negative correlations are shown as red bars below the horizontal lines. Color intensity corresponds to the slope and color saturation to *r*^2^. The observable parameters were obtained from deterministic simulations, except the latencies, which were obtained from 1000 stochastic simulations (sweeps). As initial condition, all the channel pairs were placed in the CC state. For this analysis, p_1_ and p_2_ from the deterministic simulations were used instead of f_1_ and f_2_ obtained from the *n* = 1000 sweeps. While this figure summarizes the results using the regression slope and *r*^2^, explicit plots of the natural logarithms of each marker vs. the energy change for every compound state and barrier can be generated by the MATLAB code deposited on Zenodo.

In Figure 3, only the energy of the composite CO/OC states was changed. However, an interaction between the channels may involve changes in the free energies of other composite states and/or barriers between composite states. To explore systematically the effects of such changes, a sensitivity analysis was conducted in Figure 4 for the model presented in Figure 3, in which these free energies were varied individually. This involved three composite states (CC, CO [identical to OC and hence referred to as CO/OC or simply CO] and OO), and two barriers (between CC and CO and between CO and OO), given the symmetry of the model (Figure 1A). Ion current parameters (maximal current, maximal activation slope, mean and median latencies of successive openings/closings, peak p_1_, peak p_2_ and time of peak p_1_ (p_1_ and p_2_, the expectation values for f_1_ and f_2_ were obtained from deterministic simulations) were then correlated to the free energy changes (Figure 4). Positive values (green bars) indicate that raising the free energy of a state/barrier increased the corresponding parameter; conversely, negative values (red bars) indicate that raising the free energy of a state/barrier decreased the corresponding parameter. For example, macroscopic activation rate (maximal activation slope, second column in Figure 4) was accelerated by raising the energy of the composite CC state because it destabilized this state and precipitated the opening of either one of the channels (transition to CO/OC). Conversely, raising the CC-CO barrier (jointly with the CC-OC barrier) slowed macroscopic activation because it opposed the exit from the CC state. Regarding the latencies of successive openings and of successive closings, Figure 4 shows that raising the energy of the composite CO/OC states strongly decreased these latencies, consistent with the histograms in Figure 3C and with the hypothesis that destabilizing the CO/OC states tends to synchronize openings and closings. These latencies were however also modulated (although, in absolute value, to a lesser extent) by changing the energies of the barriers. Raising the energy of the CO/OC states also increased peak p_2_ while decreasing peak p_1_. From the five interventions on states/barriers shown in Figure 4, raising the CO/OC states was the most compatible with coupled gating, the increase of f_2_ and the decrease of f_1_ without large macroscopic current changes, as reported by Clatot et al. (2017) for Na^+^ channel dimers. However, other changes in the global energy profile may be involved. Furthermore, the maximal current also correlated slightly negatively with the energy of the CO/OC states (Figure 3B).

The same analysis was conducted for a single-channel opening rate of 2 ms^–1^ and closing rate of 1 ms^–1^ (Supplementary Figure S2). The results were essentially similar, with the exception that the energy of CO/OC state and maximal current were positively correlated, as also visible in Supplementary Figure S1B.

Altogether, these results suggest that an increased free energy of composite CO states underlies the interaction between Na^+^ channels, although other mechanisms may exist. However, the 2-state C-O channel model is incomplete because Na^+^ channels also exhibit inactivated states. We therefore extended our study to Markovian models incorporating inactivation.

### Lessons From a Composite Pair of 3-State Markov Models

**FIGURE 5:**
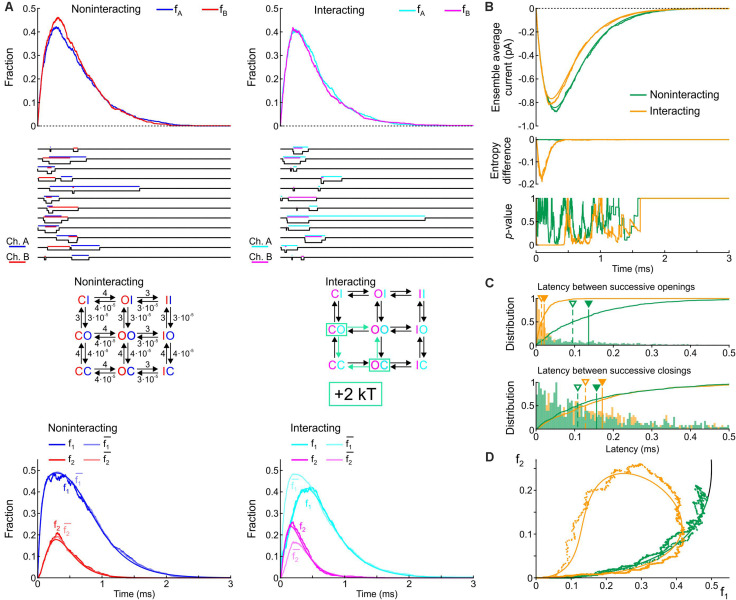
Simulated gating behavior of a pair of 3-state channels (C: closed ↔ O: open ↔ I: inactivated) in the absence of interaction and upon raising the energy of the composite CO and OC states by 2 kT. Same protocol, analysis and panel layout as in Figure 3.

In this next step, we implemented a single-channel model with three states: closed, open and inactivated (C, O, and I), with an activation rate (C→O) of 4 ms^–1^ and an inactivation rate (O→I) of 3 ms^–1^. The reverse rates were set to 10^–5^ times these values and had a negligible influence on the activation/inactivation processes. As initial condition, the channels were placed in the C state. Compound models of two channels were then constructed without interaction and with an interaction mediated by a 2 kT increase of the energy of the CO/OC states. Figure 5 shows corresponding diagrams, fractions/probabilities, individual simulated sweeps, ensemble average currents and further parameters in the same manner as Figure 3.

Without interaction, the model yielded a rapid activation followed by slower inactivation, with a peak open channel fraction (f_A_, f_B_) near ∼0.4 at time ∼0.25 (Figure 5A, *top*), generating an ensemble average current (Figure 5B, *top*) similar to experimental recordings of human cardiac Na^+^ currents at physiological temperature (Keller et al., 2005). Corresponding single-channel sweeps (Figure 5A) show that the openings and closings of the two non-interacting channels were uncorrelated, with longer periods with one channel open and shorter periods with both channels open. With the interaction, the sweeps already reveal that the channels tend to synchronize their openings but not their closings. The bottom panels of Figure 5A shows that the interaction decreased f_1_. The interaction also increased f_2_, but only during the activation phase. Furthermore, for the interacting channels, the interaction was again reflected by f_1_ < $\bar{{\text{f}}_{1}}$ and by f_2_ > $\bar{{\text{f}}_{2}}$ (especially during activation and near the peak), while for the non-interacting channels, f_1_ ≈ $\bar{{\text{f}}_{1}}$ and f_2_ ≈ $\bar{{\text{f}}_{2}}$. The top panel of Figure 5B shows that the interaction slightly decreased peak average current and the time of peak, without major changes in activation and inactivation kinetics. The entropy difference reached a minimum near ∼-0.16 early during activation but returned to 0 during the inactivation phase. Moreover, the *p*-value of Fisher’s exact test, was close to 0 only during activation and near the peak of the current. Without interaction, the entropy difference remained 0 and Fisher’s test showed no significant interdependence, as expected. These observations indicate that the interaction essentially affected activation rather than inactivation. The histograms in Figure 5C show that the latency between successive openings was decreased by more than 5-fold by raising the energy of the composite CO states by 2 kT; however, this interaction slightly prolonged the latency between successive closings, which contrasts with experimental reports (Clatot et al., 2017) and with the 2-state model (Figure 3C). This suggests that other types of interaction between Na^+^ channels must be involved. Nevertheless, in the f_2_ vs. f_1_ plot (Figure 5D), raising the energy of the composite CO states replicated the clockwise loop with a decaying late part along the theoretical expectation curve (see Figure 2), while, without interaction, the trajectory collapsed on the theoretical expectation.

Na^+^ channels can undergo closed-state inactivation and closed-state recovery from inactivation (Nakajima et al., 2019). Therefore, we repeated these simulations and analyses for a “triangular” rather than “linear” 3-state COI model with an activation rate (C→O) of 3 ms^–1^, an open-state inactivation rate (O→I) of 3 ms^–1^ and a closed-state inactivation rate (C→I) of 3 ms^–1^. The respective reverse rates were set to comparatively very small values of 3⋅10^–5^ ms^–1^, 3⋅10^–5^ ms^–1^ and 3⋅10^–10^ ms^–1^ (satisfying microscopic reversibility). The compound model, with vs. without an increase by 2 kT of the composite CO/OC states, yielded similar results (Supplementary Figure S3) as the “linear” 3-state COI model.

**FIGURE 6:**
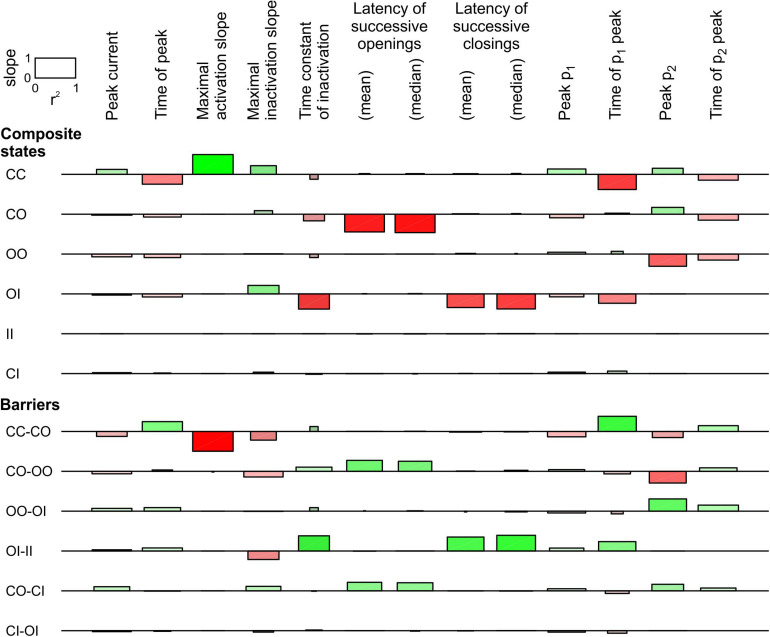
Sensitivity analysis for the pair of 2-state channels used in Figure 5 (linear COI model, C: closed ↔ O: open ↔ I: inactivated). Same protocol, analysis and layout as in Figure 4. The rate constants for the non-interacting single channels are shown in Figure 5A. Additional observable parameters (labels on top) are: peak current, maximal inactivation slope, time constant of inactivation, and time of peak p_2_ (from deterministic simulations).

For the linear COI model, we proceeded with a sensitivity analysis similar to that in Figure 4. This analysis, shown in Figure 6, involved varying the energy of six composite states and six barriers between composite states. Additionally, the sensitivity was ascertained for the time of peak current, the maximal slope of macroscopic inactivation, the time constant of macroscopic inactivation, and the timing of peak p_2_. Raising the energy of the composite CO states led to a decrease of peak p_1_ and an increase of peak p_2_, and to a prominent decrease of the latency of successive openings. It also delayed the time to peak p_1_ and shortened the time to peak p_2_. This intervention did not affect the latency of successive closings, while it accelerated inactivation without major change of other observable parameters. Regarding the effects on p_1_ and p_2_, the composite states and barriers for which energy shifts led to opposite changes in peak p_1_ and p_2_, were, next to CO, the composite state OO and the barriers CO-OO and OO-OI. Changing the energy of the OO state and the OO-OI barrier had however no influence on the latencies. Nevertheless, lowering the energy barrier CO-OO induced effects on p_1_, p_2_, and on the latency of successive openings that were similar to raising the energy of the state CO, suggesting that changes in this barrier are also a plausible interaction mechanism. Regarding the latency of successive closings, this parameter was only shortened by raising the energy of the state OI and by lowering the barrier OI-II, which precipitates inactivation of one channel if the other is already inactivated.

In summary, these observations indicate that changes to more than one composite state or barrier are necessary to account for the synchronization of both openings and closings. Our analysis identifies the energies of the composite states involving O (CO, OO, and OI) to account for coupled gating. It also pinpoints the energy barriers between OO and other composite states (CO-OO and OO-OI), as well as the barrier OI-II, as possible coupled gating mechanisms.

In Supplementary Figure S4, we conducted the sensitivity analysis for the triangular COI model of Supplementary Figure S3. The results were essentially similar to those for the linear COI model. The analysis involved three additional barriers, CC-CI, CO-IO, and CI-II, of which none produced opposed effects on p_1_ and p_2_.

### Sensitivity Analysis for a Full Cardiac Sodium Channel Model

During activation, Na^+^ channels undergo several conformational changes before finally arriving to the open conducting state (Hille, 2001). Na^+^ channels also exhibit different time courses of inactivation and recovery from inactivation, which can be explained by different inactivated states at different inactivation depths (Clancy and Rudy, 1999; Bondarenko et al., 2004). The 3-state model studied above is thus incomplete and must be complemented with additional closed and inactivated states. One previously published human cardiac wild-type Na^+^ channel model considering these features is the model of Clancy and Rudy (1999). This 6-state model (Figure 1A) incorporates three closed states (C3, C2, and C1), one open state (O), and two inactivated states (IF: fast inactivated and IS: slow or deep inactivated). The model also accounts for closed-state inactivation (C1 to IF).

**FIGURE 7:**
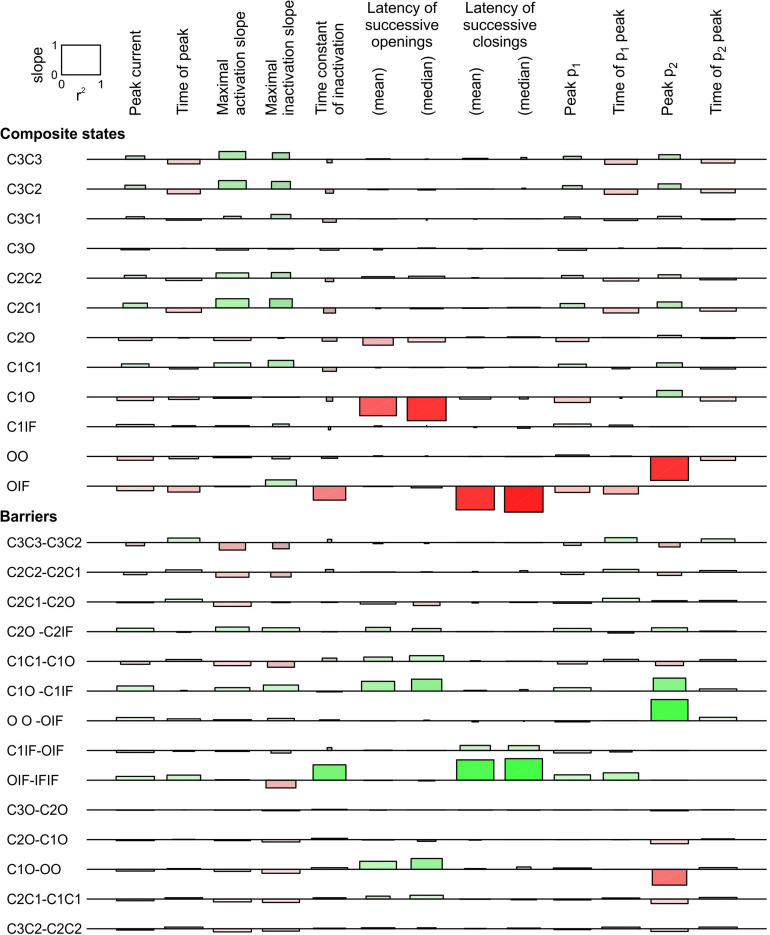
Sensitivity analysis for a pair of 6-state Clancy–Rudy model channels (Clancy and Rudy, 1999) for a voltage step to -20 mV. Same analysis and layout as in Figure 6. As initial condition, all channels were placed in the C3 state. For the simulations, the rate constants at -20 mV were used. Simulations were run for 5 ms for this analysis.

In Figure 7, we conducted a sensitivity analysis for a pair of Clancy-Rudy model channels subjected to an activating voltage step to -20 mV. The complete analysis included 21 possible composite states and 36 possible energy barriers. In Figure 7 only the states and barriers related with at least one change with a regression slope > 0.1 (in absolute value) are shown. Changing the energies of other composite states and barriers (most involving the IS state) affected the investigated parameters only minimally.

Regarding the composite states, raising the energies of C3O, C2O, and C1O increased peak p_2_, decreased peak p_1_, and shortened the latencies between successive openings, without affecting the latencies between successive closings. This is consistent with the results obtained with the 3-state model (Figures 5, 6). These effects were largest for C1O and smaller for C2O and C3O. Lowering the energy of OO strongly increased peak p_2_ because it rendered this composite state more stable; however, this intervention did not affect the latencies. Raising OIF was the only composite state energy modulation that strongly shortened the latency between successive closings, but it also strongly accelerated macroscopic inactivation.

Regarding the barriers between composite states, lowering C1O-OO tended to synchronize openings, increased peak p_2_ and decreased peak p_1_. This is again consistent with the 3-state model. Lowering C2O-C1O and C3O-C2O had the same effects, although smaller. Synchronization of openings was also strongly potentiated by lowering C1O-C1IF, although this intervention decreased p_2_. Raising the OO-OIF barrier increased p_2_, as it slowed down the exit from the OO state. Finally, among all barriers, only lowering OIF-IFIF exerted a substantial synchronization of closings. This goes along with the notion that a channel in the IF state precipitates the inactivation of the other channel if it is in the open state, leading to coupled closing.

This analysis thus identifies the composite states C3O, C2O, C1O, OO, and OIF as well as the barriers C3O-C2O, C2O-C1O, C1O-OO, OO-OIF, OIF-IFIF, and C1O-C1IF as the most likely candidates for which the channel-channel interaction modulates their free energy.

### Systematic Exploration of Interaction Profiles

Since none of the individual energy changes of composite states or barriers replicated both the synchronization of openings and closings together with an increase in p_2_ and a decrease in p_1_, we applied several changes in combination. We also accounted the observation of Clatot et al. (2017) that disrupting cardiac Na^+^ channel interaction and coupled gating with difopein did not change the ensemble average current.

**FIGURE 8:**
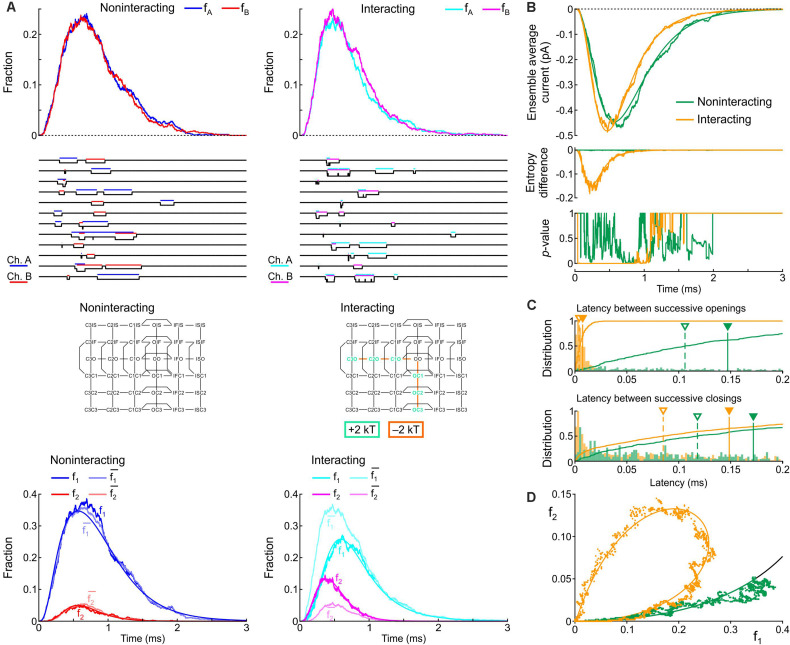
Simulated gating behavior of a pair of 6-state Clancy–Rudy model channels (Clancy and Rudy, 1999) in the absence of interaction and upon raising the energies of the composite C3O, C2O and C1O states by 2 kT, and lowering the energies of the C3O-C2O, C2O-C1O, and C1O-OO barriers by 2 kT (Interaction I; color-coded diagram). Same analysis and panel layout as in Figure 5. As initial condition, all channels were placed in the C3 state. For the simulations, the rate constants at -20 mV were used.

Firstly, we varied the energies of the following composite states and barriers as follows: (i) the energies of the C3O, C2O, and C1O states were raised jointly by 0, 1, or 2 kT and (ii) the energies of the C3O-C2O, C2O-C1O, and C1O-OO barriers were lowered jointly by 0, 1, or 2 kT. The combination that induced the largest increase of peak p_2_ and the least changes to the average current in terms of peak, time to peak and time constant of inactivation was a raise of C3O, C2O, and C1O by 2 kT and a lowering of C3O-C2O, C2O-C1O, and C1O-OO by 2 kT. Hereafter, we refer to this interaction profile, illustrated in Figure 8, as Interaction I.

Figure 8A shows that Interaction I clearly increased f_2_ and decreased f_1_, with f_2 >_ $\bar{{\text{f}}_{2}}$ and f_1 <_ $\bar{{\text{f}}_{1}}$, without manifest change in the peaks of f_A_ and f_B_. The individual sweeps show that channels exhibit coupled openings and closings, while the channels open and close essentially separately without interaction. The interaction was also reflected by the negative entropy difference, by the *p*-value close to 0 during and shortly after the peak (Figure 8B), and by the large clockwise loop in the f_1_-f_2_ diagram (Figure 8D). Moreover, interaction I drastically reduced the latency between successive openings, but only modestly decreased the latency between successive closings (Figure 8C). The histogram for the successive closings exhibited a long tail; nevertheless, a substantial fraction of the latencies was apparent below 0.15 ms (yellow part of the histogram). We thus considered Interaction I as an interaction compatible with experimental observations.

However, as visible in Figure 8B, Interaction I slightly increased peak current, accelerated activation, shortened time to peak and accelerated inactivation. These changes ranged up to ∼20% and thus possibly escape detection in experiments due to measurement error and biological variability. These changes in macroscopic current properties were nevertheless large enough to motivate us to search further for possible interaction profiles.

**FIGURE 9:**
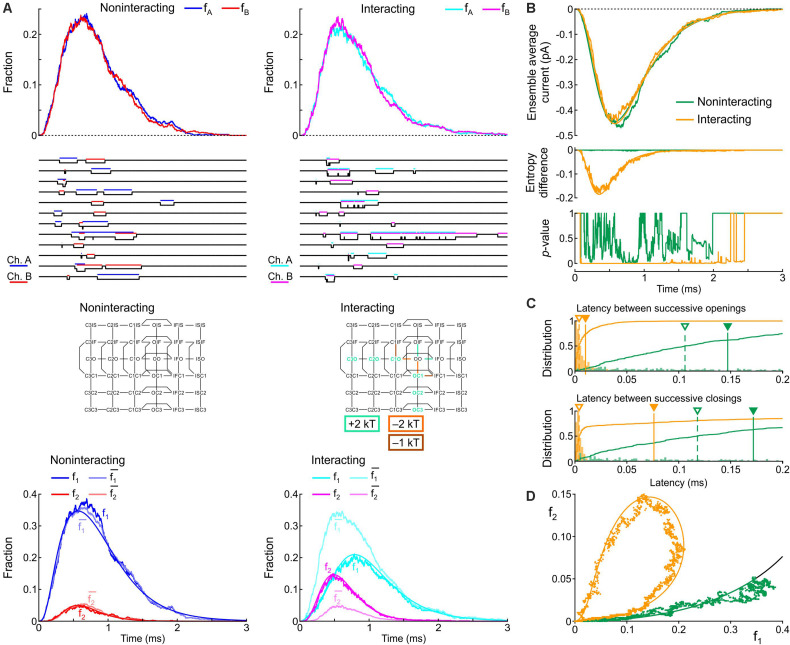
Simulated gating behavior of a pair of 6-state Clancy–Rudy model channels (Clancy and Rudy, 1999) in the absence of interaction and upon raising the energies of the composite C3O, C2O and C1O states by 2 kT, lowering the energy of the C1O-OO barrier by 2 kT, and lowering the energy of the C1O-C1IF barrier by 1 kT (Interaction II; color-coded diagram). Same protocol, analysis and panel layout as in Figure 8.

Secondly, based on the identification of most likely candidates in the previous section, the energies of the following composite states and barriers (or sets of composite states and barriers) were systematically varied as follows. (i) C3O, C2O, and C1O were varied jointly by 0, +1, or +2 kT; (ii) OIF was varied by −1, 0, or +1 kT; (iii) OO was varied by −1, 0, or +1 kT; (iv) C1O-OO was varied by −2, −1, or 0 kT; (v) C3O-C2 and C2O-C1O were varied jointly by −2, −1, or 0 kT; (vi) OIF-IFIF was varied by −1, 0, or +1 kT; (vii) OO-OIF was varied by 0, +1 or +2 kT; and (viii) C1O-C1IF was varied by −1, 0, or +1 kT. This resulted in 3^8^ = 6561 possible combinations, which were all simulated. From these combinations, we disregarded those for which the peak current, the time of peak current, or the time constant of inactivation differed by more than 5% from the control simulation of two non-interacting channels. In the remaining subset, we retained combinations that led to a > 90% decrease of the median latency between successive openings and successive closings, a > 2-fold increase of peak p_2_, a > 30% decrease of peak p_1_, and a large clockwise loop trajectory in the p_2_ vs. p_1_ plot initially above and then along the curve expected for non-interacting channels. The combination meeting these criteria was obtained by raising C3O, C2O, and C1O by 2 kT, lowering the C1O-OO barrier by 2 kT, and lowering the barrier C1O-C1IF by 1 kT (Figure 9). Hereafter, we refer to this interaction profile as Interaction II.

Figure 9 shows the effects of Interaction II in the same manner as Figure 8. With Interaction II, f_A_ and f_B_ almost followed the same time course as without interaction (Figure 9A) and the ensemble average current was almost the same (Figure 9B). At the single-channel level, the interacting channels exhibited coupled openings and closings (Figure 9A), documented by the histograms of the latencies (Figure 9C). In the histogram of the latency between successive closings, Interaction II now clearly decreased the median by more than 10-fold, while it decreased the mean by only ∼30%, because the histograms exhibited long tails. The behavior of f_1_, f_2_, $\bar{{\text{f}}_{1}}$ and $\bar{{\text{f}}_{2}}$ (Figure 9A), the entropy difference, the *p*-value (Figure 9B), and the clockwise f_2_ vs. f_1_ loop (Figure 9D) were otherwise similar to those with Interaction I.

To validate our computational approach, we compare in Supplementary Figures S5, S6 the L0, L1 and L2 counts and corresponding deterministic model expectations simulated using the matrix exponential algorithm and Gillespie’s algorithm for the pair of wild type Clancy-Rudy models with Interaction II. Both algorithms produced similar results. In Supplementary Figure S7, we show histograms for 5 realizations of the simulation with the wild-type Clancy-Rudy model pair without interaction and with Interaction II. Although not exactly identical due the stochastic nature of the simulations, the histograms all have a similar aspect. In Supplementary Figure S8, we repeated the same simulation but with a 10 times shorter time step and narrower bins; this Figure shows that the histograms do not exhibit peaks or modes but are monotonically decreasing.

**FIGURE 10:**
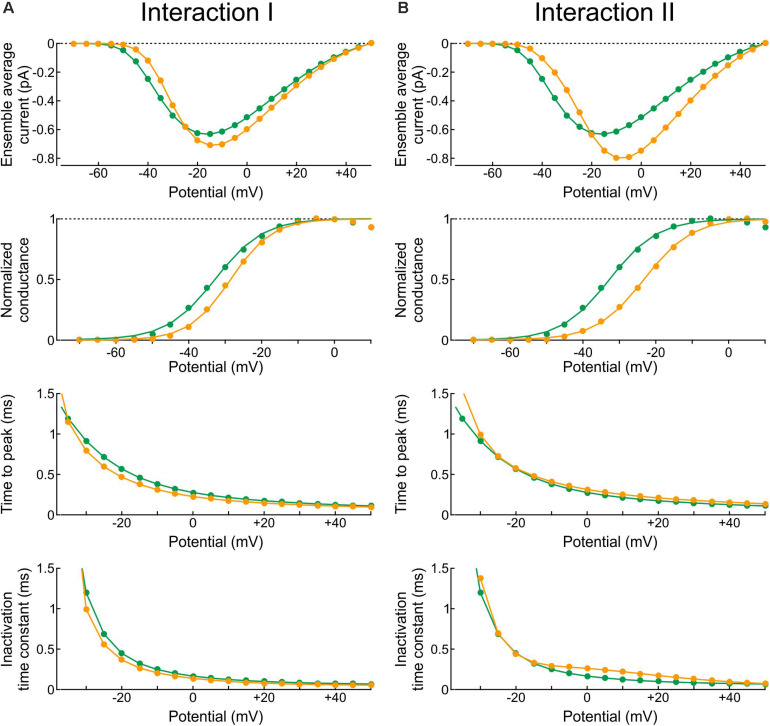
Reconstructed peak ensemble average current vs. voltage relationships (I-V curves, *top row*), normalized conductance (*second row*), time to peak (*third row*), and inactivation time constant (*bottom row*). **(A)** In the presence of Interaction I (orange) vs. no interaction (green). **(B)** In the presence of Interaction II (orange) vs. no interaction (green). The data were obtained using deterministic simulations.

In the Clancy-Rudy model, all rate constants are functions of membrane potential V. Therefore, we conducted simulations of voltage clamp activation protocols by stepping V at time 0 to values ranging from -70 mV to +60 mV in steps of 5 mV. Figure 10 shows the resulting peak ensemble average current (I–V curves), the normalized conductance, the time to peak, and the inactivation time constant plotted against V with Interaction I (Figure 10A) and Interaction II (Figure 10B) vs. without interaction. The normalized conductance curves were fitted with the function g_norm_ = 1/(1 + exp((V_1__/__2_−V)/k)), with V_1__/__2_ being the half activation potential and k the slope factor.

Both interactions changed the overall shape of the I-V curve, making it steeper with a higher overall peak. Since the interaction models were adjusted to produce a similar peak at −20 mV, the curves crossed over near −20 mV. In terms of normalized conductance, Interaction I slightly shifted V_1__/__2_ from −32.7 to −28.5 mV and slightly decreased k (from 6.6 to 5.7 mV), whereas Interaction II shifted V_1__/__2_ from −32.7 to −23.2 mV without changing k (6.6 mV). Interaction I slightly shortened time to peak for V between −5 and 40 mV, while Interaction II slightly delayed it for V above −5 mV; finally, the inactivation time constant was slightly shortened by Interaction I and lengthened by Interaction II between −10 and 40 mV.

In brief, the changes in macroscopic current parameters with Interaction I were moderate and still within the range of biological variability observed in experiments. However, the changes with Interaction II were more pronounced. This highlights the difficulty of obtaining an energy interaction profile that would lead to coupled openings and closings without affecting macroscopic current parameters at all possible potentials.

### Interactions Can Contribute to the Negative Dominance of Cardiac Na^+^ Channel Variants

Variants of the gene *SCN5A* encoding the α-subunit of the cardiac sodium channel Na_v_1.5 can cause cardiac arrhythmias such as Brugada syndrome or long-QT syndrome type 3 (Lieve and Wilde, 2015; Veerman et al., 2015). To explore how channel-channel interactions influences gating and macroscopic Na^+^ currents in the presence of a channel variant, we used our coupled channel model with Interactions I and II.

We considered the variant p.L325R, which was first described in a patient presenting with Brugada syndrome during episodes of fever (Keller et al., 2005). As hallmark of negative dominance, when HEK cells are transfected with equal amounts of DNA coding for WT and variant p.L325R channels, the resulting macroscopic current is less than half (about 25%) of the current generated by cells transfected with the corresponding amount of WT DNA. Both WT and variant channels are trafficked to the membrane, suggesting that the mechanism of negative dominance involves phenomena occurring at the cell membrane and presumably direct channel interactions (Keller et al., 2005; Clatot et al., 2012, 2017, 2018).

**FIGURE 11:**
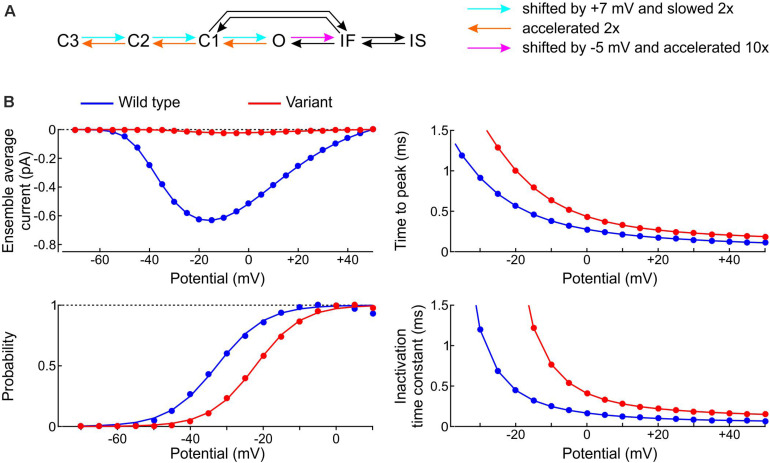
Model of the variant p.L325R channel. **(A)** Diagram showing the modifications of the rate constants of the Clancy–Rudy model (color legend). **(B)** Peak ensemble average current (I–V curve), time to peak, normalized conductance (activation curve), and inactivation time constant vs. potential for the p.L325R channel (red) vs. the WT channel (blue).

We started by formulating a single-channel model of the p.L325R variant by modifying the rate constants of the Clancy-Rudy model, as illustrated in Figure 11A. In patch clamp experiments, the most salient biophysical properties of the macroscopic p.L325R current are a severalfold decrease in peak current, a shift of the activation curve by ∼10 mV to more positive potentials, a slight increase of the time to peak and a doubling of the inactivation time constant at −20 mV (Keller et al., 2005). To simulate these features, we shifted the opening rates by +7 mV and slowed them by 50%, doubled the deactivation rates, and shifted the rate of fast inactivation by −5 mV while scaling it by a factor 10 (Figure 11A; the rate IF→O was adjusted in agreement with microscopic reversibility). In line with experiments, this resulted (see Figure 11B) in a > 10-fold reduction in peak Na^+^ current, an increase of time to peak by ∼30%, a shift of the V_1__/__2_ of activation by 10.6 mV (from −32.7 to −22.1 mV; k was only minimally affected: 6.3 mV vs. 6.6 mV), and a 2–3-fold increase of the inactivation time constant.

**FIGURE 12:**
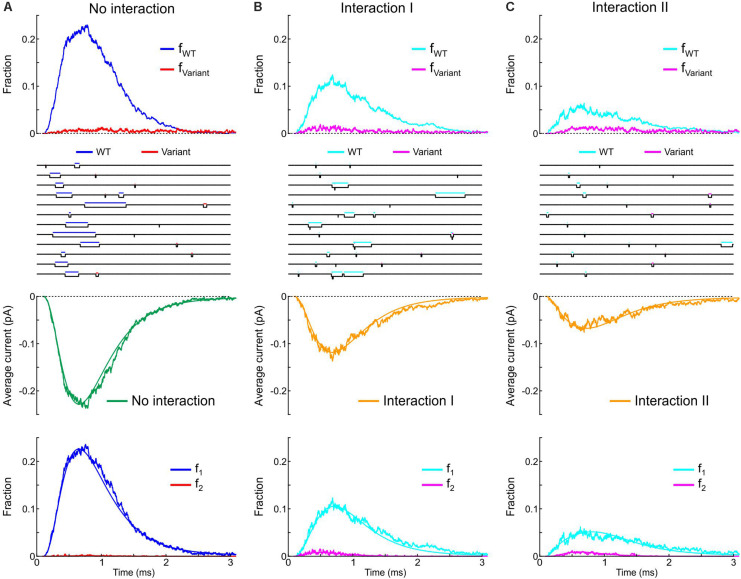
Simulated gating behavior of a channel pair consisting of a WT Na^+^ channel (nominal Clancy–Rudy model) and a p.L325R variant Na^+^ channel without interaction **(A)** and in the presence of Interaction I **(B)** and Interaction II **(C)**. Stochastic simulations (*n* = 1000 sweeps) were conducted for a voltage step to -20 mV. As initial condition, all channels were placed in the C3 state. *First row:* Fractions f_WT_ and f_Variant_ of the individual channels being open in the *n* sweeps. *Second row:* Simulated sweeps. The simulated current is represented in black; the intervals during which the channels were open are marked by colored overbars. *Third row:* Ensemble average current (the same single-channel conductance was assumed for both channels). *Fourth row:* Fractions of sweeps f_1_ and f_2_ with one channel (irrespective of which one) or two channels open. Smooth curves were obtained from deterministic simulations.

Next, we incorporated the variant p.L325R Na^+^ channel model into our channel pair framework and simulated the behavior of a heterodimer consisting of one WT and one p.L325R variant channel, first without any interaction (Figure 12A) and then with Interaction I (Figure 12B) and Interaction II (Figure 12C), for a voltage step to −20 mV. At this potential, the interactions only slightly change the average current for a dimer of WT channels (Figures 8, 9).

Without interaction (Figure 12A), the WT channel gated normally, whereas the variant channel activated slowly and inactivated quickly, exhibiting only scarce and short-lived openings. Accordingly, the open fraction for the WT channel (f_WT_) exhibited a normal time course, while the open fraction for the variant channel (f_Variant_) remained near 0. The WT channel therefore essentially determined the ensemble average current (assuming that both channels produce the same unitary current of −1 pA). Furthermore, f_2_ remained near 0, while f_1_ was essentially determined by the WT channel openings. With Interaction I (Figure 12B), f_WT_ was clearly reduced by the interaction with the variant and the WT channel openings were on average shorter. The variant channel still opened scarcely and during short times, with f_Variant_ remaining near 0. The interaction therefore reduced the ensemble average current (peak reduced by ∼50%). Although the interaction slightly increased f_2_, this increase was not sufficient to compensate for the decrease of f_WT_ and f_1_. With Interaction II (Figure 12C), these effects were even more prominent, with a ∼70% decrease of peak average current compared to the model without interactions.

Thus, in our model, the interactions between Na^+^ channels lead to coupled gating and an increase in f_2_ for a normal WT channel pair, but result in a strongly negative impact of the variant on the WT channel for a heterologous WT-variant pair. The analyses involving the calculation of $\bar{{\text{f}}_{1}}$ and $\bar{{\text{f}}_{2}}$, the entropy difference, the histograms of latencies, the use of Fisher’s or χ^2^ tests as well as the representation of f_2_ vs. f_1_ do not apply in this case, because the channels are not identical. However, in conventional single-channel recordings, the channels would be indistinguishable (if the unitary currents are the same), and only f_1_ and f_2_ (but not f_WT_ and f_Variant_) could be obtained experimentally.

**FIGURE 13:**
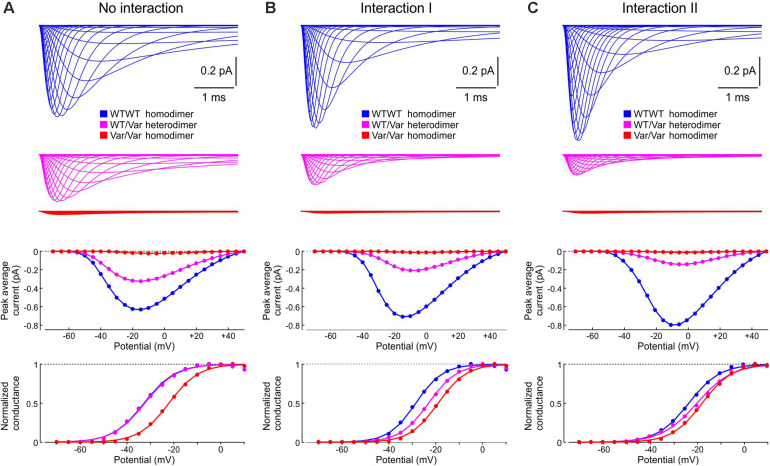
Simulated sets of Na^+^ currents, current-voltage relationships and activation curves that would be obtained by an activation protocol for a WT/WT homodimer (blue), a WT/p.L325R variant heterodimer (magenta) and a p.L325R/p.L325R homodimer (red). **(A)** Without interaction. **(B)** With Interaction I. **(C)** With Interaction II. Deterministic simulations were conducted for voltage steps to values from −70 mV to 50 mV in 5 mV increments. As initial condition, all channels were placed in the C3 state. *Top:* Current traces. *Middle:* Peak current-voltage relationships. *Bottom:* Activation curves.

Finally, in Figure 13, we examined how the interactions between WT and p.L325R variant channels affect sets of currents, current-voltage relationships, and activation curves that would typically be obtained using whole-cell patch clamp experiments by an activation protocol. Without interaction (Figure 13A), the current generated by heterodimers (WT/Var) was about half of the current generated by the WT/WT homodimers, while the current produced by the variant homodimers was very small. The activation curve for the heterodimer situation (WT/Var) overlapped with that of the WT/WT because the variant channel hardly produced any current. With Interaction I (Figure 13B), the current generated by heterodimers was clearly less than half of that generated by WT/WT dimers, and the activation curve of the WT/Var was between those of the homodimers. With Interaction II (Figure 13C), the WT/Var current was reduced even further to ∼20% of the WT/WT current. These results indicate that interactions between channels can contribute to the negative dominance of certain Na_v_1.5 variants.

## Discussion

We developed a framework combining two Markovian ion channel models into a compound model to examine the consequences of interactions between channels on their microscopic and macroscopic kinetics. In such a compound model, every state of the first channel can be associated with any state of the second. The model then implements interactions as free energy changes of composite states and barriers between composite states. We proceeded with an incremental approach with channel models of increasing complexity. In the compound of two 2-state closed-open models, raising the free energy of composite CO states resulted in a synchronization of individual channel openings and closings (coupled gating), suggesting that heterogeneous composite CO states are indeed less stable. In the compound of two 3-state closed-open-inactivated models, the same intervention led to coupled openings, but, based on our sensitivity analysis, other changes in the energy profile of the compound model must be introduced to obtain coupled closings. Using the Clancy-Rudy Na^+^ channel model, we evaluated a large set of energy profile variations to identify interactions that reproduced experimental observations (Clatot et al., 2017): coupled openings, coupled closings, increased sweep counts with two channels open simultaneously at a given time during activation, decreased sweep counts with only one channel open, and a clockwise loop in the f_1_-f_2_ diagram.

**FIGURE 14:**
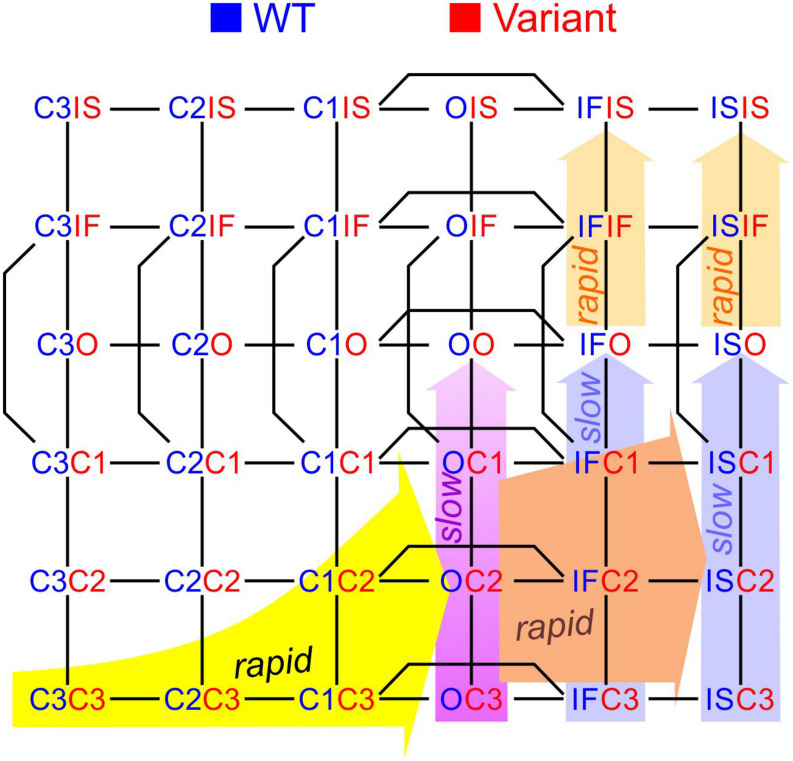
Working model to explain how interactions between a wild-type (blue state labels) and a variant p.L325R Na^+^ channel (red state labels) decrease the Na^+^ current and contribute to the negative dominance of the variant. See text for description.

Next, taking the p.L325R DN variant of Na_v_1.5 as an example, we investigated whether channel-channel interactions can directly contribute to the negative dominance of the variant over the wild type. For this, we first formulated a single-channel model of the p.L325R variant by modifying the rate constants such that the model matches experimental data (Keller et al., 2005). Then, we investigated the interactions between a WT and a variant Na^+^ channel. Our working model describing how such interactions decrease the Na^+^ current and contribute to the negative dominance of the variant is summarized schematically in Figure 14. Upon depolarization, the WT channel activates normally and rapidly from the C3 state through the C2, C1, and O state (yellow background arrow in Figure 14). Because the variant channel activates slowly, it proceeds to a much lesser extent toward its C2 and C1 states. The trajectory of the channel pair (yellow) in the two-dimensional graph of Figure 14 thus lies below the diagonal. The channel pair then arrives in a composite state with the WT channel open and the variant channel still in a C state. Because the activation of the variant is slow (purple background arrow), and now also because the interaction increases the free energy of the composite CO states, these states are less stable and thus decay rapidly into states in which the WT is inactivated while the variant is still closed (orange background arrow). Thus, the OO state is essentially bypassed. At this stage, the variant channel still slowly activates (blue background arrows), and the composite states consisting of the inactivated WT channel and the open variant channel are eventually reached. However, because inactivation of the variant is accelerated, these states are short-lived and decay quickly into the inactivated/inactivated states (amber arrows). As a net result, with interactions, the WT channel contributes less to the Na^+^ current than without interactions, while the contribution of the variant channel remains minimal.

The question remains open how other variants interacting with WT channels affect the Na^+^ current. Theoretically, the opposite phenomenon may occur, whereby a variant channel potentiates the current generated by the WT, leading to a gain of function. Conversely, certain Na^+^ channel variants that would, on their own, result in altered function, may be rescued by their interaction with the WT, as proposed recently for Na_v_1.7 (Rühlmann et al., 2020). Heterozygous carriers would then not necessarily manifest symptoms unless, for instance, the interaction is altered by a drug. Our modeling framework opens the door to investigate such possibilities.

Our approach may also be used to investigate the recently demonstrated interactions between Ca_v_1.2 calcium channels (Dixon et al., 2015; Ito et al., 2019). We underline that our approach can easily be generalized to combine two completely different types of channels, such as Na^+^ and K^+^ channels. In this setting, if recordings at the single-channel level become available in the future, our approach is useful to model and investigate gating interactions between for instance Na_v_1.5 and K_ir_2.1 channels, which are known to colocalize on the cardiac cell membrane and to form macromolecular complexes (Milstein et al., 2012; Perez-Hernandez et al., 2018). Similarly, our approach may be used to investigate interactions between Na_v_1.5 and K_v_11.1 (hERG) channels, which were shown to interact during transcription and as nascent proteins, resulting in correlated intensities of corresponding membrane currents (Eichel et al., 2019).

It would be insightful to investigate what happens if the rate coefficients of the starting Markov models are slightly different, e.g., due to differences that may arise between cellular expression systems and real myocytes, or due to differences in applied voltage. Such a study would then need to be very extensive: for the Clancy-Rudy model, 11 independent rates would have to be systematically varied, which, in combination with 15 independent energies of states and >30 possible barriers, would lead to a very large parameter space. Such a study goes beyond the scope of the present work.

### How to Quantify the Interactions Between Channels?

To analyze both experimental and simulation data, appropriate approaches are essential to ascertain whether channels interact. The statistical properties of recordings from two or several independent channels have been extensively investigated in the past (Yeo et al., 1989; Colquhoun and Hawkes, 1990; Fredkin and Rice, 1991). In subsequent work by Clatot et al. (2018), the interaction between Na^+^ channels was quantified using a procedure proposed by Chung and Kennedy (1996). However, all these methods presuppose that the behavior of the investigated channels is stationary (i.e., that a steady-state is present), which is obviously not the case for Na^+^ channels upon an activating voltage step. Chung and Kennedy’s analysis (Chung and Kennedy, 1996) furthermore presupposes that deviation from microscopic reversibility is possible, which contradicts the principle of conservation of energy.

Thus, methods taking into account the transient behavior of Na^+^ channels (or any time-dependent voltage-gated channels) should be applied. Our development of such methods was further motivated by the fact that the influence of one Na^+^ channel on another likely exhibits time-dependence; thus, quantitative measures should be functions of time rather than single scalars.

Conventional χ^2^ and Fisher’s tests typically used to test interdependence can readily be used to document the existence of an interaction. Applying these tests at individual time points permits to identify the phases during which the interaction is significant. Measures derived from information theory, such as Shannon’s entropy, can be useful in tracking the level of interaction with time. Finally, a graphical representation of f_2_ versus f_1_ is very helpful to visualize the interaction for identical channels. When we applied these approaches on Clatot’s data (2017), we found that the interaction between wild-type Na_v_1.5 channels is highly significant during activation and around the peak of macroscopic Na^+^ current, but this significance vanishes during inactivation as the entropy difference returns to 0. Interestingly, with difopein, the interaction remained, albeit to a lesser extent. Possibly, difopein disrupted only the interactions mediated by 14-3-3 but not those resulting from direct contacts between the α-subunits of the Na^+^ channels. Alternatively, due to the binding and unbinding kinetics of difopein to 14-3-3, some sweeps may be recorded with interacting channels and some without.

### Can There Be Interactions Without Macroscopic Current Changes?

Our sensitivity analysis showed that changing the energy of a composite state or a composite barrier influences most macroscopic parameters (e.g., peak, time to peak, inactivation time constant) besides microscopic parameters (e.g., latency between successive openings, peak f_2_). While some energetic changes can be compensated by others to a certain extent, the existence of an interaction profile that would not change macroscopic parameters appears extremely unexpected and fortuitous in our framework. Thus, it appears difficult to design a model of interactions that would lead to coupled openings and closings without affecting macroscopic current parameters at all potentials. This finding is at odds with the report of Clatot et al. (2017) that ensemble average currents from homomeric Na^+^ channel pairs were not affected by difopein (at steps to -20 and -40 mV). If Na^+^ channel interactions mediated by 14-3-3 truly do not modify macroscopic current parameters, one possibility to account for this would be to make the interaction energies voltage-dependent in the model. However, without sufficient experimental data to validate such simulations, we did not explore this possibility in the present work. Nevertheless, in our reconstruction of the ensemble average current from the single-channel data of Clatot et al. (2017) (Figure 2A) peak current as well as activation and inactivation kinetics appear somewhat differently in the absence vs. presence of difopein. While this may be related to biological variability or technical aspects of the experiments, this observation may also suggest that Na^+^ channel interactions modify the macroscopic current.

### Distinguishable vs. Indistinguishable Channels

When recording from two identical channels simultaneously using the patch clamp technique, it is inherently impossible to distinguish the contribution of each individual channel. This poses a great challenge in the analysis and interpretation of such recordings. Possibly, the patch clamp technique could be refined, for instance by combining it with voltage clamp fluorometry (Cowgill and Chanda, 2019) or by genetic engineering of the pore of one channel to alter its conductance to make it distinguishable. Any modification to channel structure may however affect channel function. These considerations do not pertain to two different types of channels, which can in principle be distinguished if their unitary current is different.

### Ephaptic Coupling Between Channels?

Alternatively, channel-channel interactions may be ephaptic rather than allosteric. Ephaptic coupling between cardiac cells represents a mechanism modulating and possibly supporting cardiac conduction across intercalated disks (Sperelakis and Mann, 1977; Kucera et al., 2002; Veeraraghavan et al., 2014). During ephaptic coupling, activated Na^+^ channels on one side of the disk generate a substantial current that flows radially through the narrow extracellular space within the intercalated disk. This large current, flowing through a confined space with high resistance, produces a substantial negative extracellular potential. This translates as membrane depolarization on the other side of the cleft, where it contributes to Na^+^ channel activation. Recently, we showed in computer simulations that clusters of Na^+^ channels in intercalated disks potentiate ephaptic coupling (Hichri et al., 2018). The question arises whether the current through a single open channel produces a sufficient electric potential or field to influence another channel in its immediate vicinity. For a single-channel current of -1 pA (Hille, 2001) and an intracellular/extracellular resistivity of 200 Ω⋅cm, the potential and the field can be estimated by assuming a point source/sink in an unbounded intracellular/extracellular half-space (Plonsey and Barr, 1988). At 12 nm from the channel mouth, the corresponding estimates are 27 μV and 22 V/cm, respectively. While this potential is too small to affect channel function, the corresponding field may suffice. Of note, at the level of the neighboring channel, this field would be tangential to the membrane, and how tangential fields affect ion channel function remains largely unknown.

If the interaction between channels was ephaptic rather than allosteric, another modeling approach would be required in which, for example, the transition between C2O and C1O (Figure 1A) is accelerated toward C1O and slowed toward C2O, while the other rates in the loop C2O-C1O-C1C1-C2C1 remain unchanged. This would violate microscopic reversibility (the loop would preferentially run clockwise). However, this is not at odds with the energy conservation principle, as the Na^+^ current would dissipate the chemical potential energy of the Na^+^ gradient and part of this energy would be consumed by the channel pair.

To test whether the interaction between Na^+^ channels is mediated by the electric field, one could design patch clamp experiments similar to those of Clatot et al. (2017) in which the direction of the Na^+^ current is changed from inward to outward by a suitable choice of Na^+^ concentration in the bath and pipette solutions. Then, the opposite effects should be observed, for instance, preferential openings of only one channel at a time and rarer occurrences of joint openings.

### Further Perspectives: From Molecular Structure to Function

Further mechanisms may be hypothesized to explain the differences between coupled openings of interacting dimerized channels and normal openings of single channels. For example, some conformational changes could only be possible for a dimer, and in this case, the Markov state diagram of each channel would be different when the channels are interacting. Since Na_v_1.5 α-subunits are large proteins with numerous degrees of freedom in their conformation, a representation using a Markov model with only a small number of stable states represents a reduction of the true system. Therefore, it is not impossible that the dimerization of Na^+^ channels attenuates certain states to such an extent that omitting them from the Markov model does not alter its general behavior.

Therefore, a comprehensive understanding of ion channel interaction will require using molecular structures to derive intra- and intermolecular interactions and exploring them in molecular dynamics simulations (Cournia et al., 2015; Delemotte et al., 2015). Silva et al. (2009), for instance, have derived a Markovian model of the KCNQ1 channel (underlying the cardiac slow delayed rectifier K^+^ current I_Ks_) from energy landscapes related to the movement of the voltage sensor, and used their model to evaluate the consequences of channel mutations on the action potential. More recently, Ramasubramanian and Rudy (2018) used molecular dynamics simulations to compute the energies of about 3 million possible I_Ks_ channel conformational states, building up an enormous multidimensional energy landscape. Channel gating was then simulated as a random walk through this landscape. Recently, the integration of atomistic molecular dynamics into electrophysiological modeling was used to predict the effects of drug toxicity on K_v_11.1 (hERG) channels (Yang et al., 2020). In such frameworks, interactions between channels can be explored by adding a Hamiltonian term to the energy profile, as commonly done in physics for multi-body dynamic problems. As the structure of the Na_v_1.5 channel is presently established (Li et al., 2019; Jiang et al., 2020), such approaches can in the future also be applied to this channel. Furthermore, molecular studies of channel dimerization and its allosteric interactions with other channels and proteins could be conducted, as done for instance for the epidermal factor growth receptor (Tsai and Nussinov, 2019). However, such studies require large computational resources. Hence, our simpler approach based on the composition of Markov models may provide initial insights to orient further research.

Our analysis of homodimer behavior is based on the assumption that both channels in the dimer are identical, such that p_A,open;B,shut_ = p_A,shut;B,open_. The question then arises whether this is also true in the presence of β subunits, which may not necessary be the same for each channel. These β subunits may also break the symmetry illustrated in Figure 1B. Concerning Na_v_1.5, we note that according to Jiang et al. (2020), β subunits increase the expression of α subunits at the membrane but they do not stably associate with Na_v_1.5 and they do not change the biophysical properties of whole cell Na^+^ currents (Makita et al., 1994; Qu et al., 1995). This suggests that β subunits do not influence the function of Na_v_1.5 channels. However, whether β subunits affect microscopic single Na_v_1.5 channel behavior is not yet fully elucidated.

### Limitations

Even though we can mimic experimental results with our proposed model to a certain extent, our model can still be refined. Because only little experimental data are available, our study is essentially exploratory rather than predictive. We did not investigate in detail the repercussion of channel-channel interactions on other processes such as deactivation and recovery from inactivation. However, to our knowledge, there are, to date, no corresponding data available at the single-channel level, and such data would be essential to develop and corroborate our modeling.

In our work, we used the Clancy and Rudy model (Clancy and Rudy, 1999), although many other Markovian Na^+^ channel models have since been developed (Irvine et al., 1999; Bondarenko et al., 2004; Moreno et al., 2011; Balbi et al., 2017; Asfaw and Bondarenko, 2019). It was not our goal to test or to compare all these models, but rather to demonstrate how one can combine Markovian models and make them interact to gain insight into channel function.

It must also be underlined that our coupled channel models are characterized by a large number of possibilities to integrate a change of free energy. For a single-channel model with N states, this number scales roughly proportionally to N^2^. For a large N, this would probably preclude an accurate identification of all possible parameters (a task that is already difficult for a single-channel model, Fink and Noble, 2009). Moreover, parameter sets that match experimental observations may not be unique, and different energy profile modifications may lead to the same microscopic and macroscopic behaviors. A further limitation is that our sensitivity analyses are linear, and thus represent a linear approximation. It is therefore possible that changing energies by larger amounts would lead to different states/barriers becoming dominant.

Finally, while our approach could in principle be extended to large clusters of Na^+^ channels, it may lead to very large matrices that computationally may become less tractable. For this purpose, phenomenological approaches to model channel cooperativity may be an advantage (Naundorf et al., 2006; Pfeiffer et al., 2020).

In any case, more electrophysiological recordings at the single-channel level over a broad range of voltages will be needed to ascertain in more detail how Na^+^ channel interactions depend on voltage and to fully evaluate the importance and the consequences of Na^+^ channel interactions in health and disease.

## Conclusion

Taken together, our results enrich the notion that Na^+^ channels dimerize and interact, and provide new insights into modeling Na^+^ channel behavior. The study of interactions between ion channels is an emerging field, and understanding the underlying mechanisms in association with future experimental studies will permit to develop better approaches to treat patients with congenital arrhythmia syndromes.

## Data Availability Statement

The datasets presented in this study can be found in online repositories. The names of the repository/repositories and accession number(s) can be found below: Zenodo, doi: 10.5281/zenodo.4064027.

## Author Contributions

JK conceived and designed the study. EH, ZS, and JK wrote the computer code, ran simulations, and analyzed the data. JK and ZS drafted and revised the manuscript. All the authors contributed to the interpretation of the data and to a critical review of the manuscript for important intellectual content, and approved the final version of the manuscript.

## Conflict of Interest

The authors declare that the research was conducted in the absence of any commercial or financial relationships that could be construed as a potential conflict of interest.
