# Supplementary material for: Modeling the Interactions Between Sodium Channels Provides Insight Into the Negative Dominance of Certain Channel Mutations
Source: Front Physiol. 2020 Nov 5;11:589386. doi: 10.3389/fphys.2020.589386 (PMC7674773; doi:10.3389/fphys.2020.589386)
Supplement: Supplementary file 1 [file Data_Sheet_1.docx]

Supplementary Material

Modeling the interactions between sodium channels provides insight into the negative dominance of certain channel mutations

Echrak Hichri, Zoja Selimi and Jan P. Kucera

# Table of contents

Analysis of L0, L1 and L2 counts using contingency tables page 2

Illustration of the Kronecker sum for two 2-state C↔O Markovian models page 6

Operators **O**_A_ and **O**_B_ to extract the state occupancy

probability vectors **p**_A_ and **p**_B_ from **p**_AB_:

example for a composite model of two 2-state channels page 9

Microscopic reversibility in composite models page 10

Example operations of changing the free energies of states

and of barriers between states page 12

Supplementary Figure S1 page 15

Supplementary Figure S2 page 16

Supplementary Figure S3 page 17

Supplementary Figure S4 page 18

Supplementary Figure S5 page 19

Supplementary Figure S6 page 20

Supplementary Figure S7 page 21

Supplementary Figure S8 page 22

**Analysis of L0, L1 and L2 counts using contingency tables**

At a given time point during a voltage clamp step protocol, two channels can be found both shut (non-conductive), one shut and the other open, or both of them open (conductive).

Considering an experiment consisting of *n* sweeps recorded under stable conditions with the same experimental protocol, and examining the sweeps at a given time point after the voltage step, the number of sweeps with zero, one or two channels open are denoted by L0, L1 and L2, respectively, as done by Clatot et al. (2017).

If the two channels, labeled A and B, are identical and if only one channel is open, it is then impossible to distinguish in a patch clamp experiment which of the two channels is open. However, because the channels are identical, the probability that it is either A or B that is open is 0.5:

P(A open | one channel open) = P(B open | one channel open) = ½.

We underline that this equal probability of A or B being open is not only valid for two identical noninteracting channels, but also for two identical interacting channels as long as the action of A on B is the same as the action of B on A.

The L0, L1 and L2 counts can be represented as a contingency table (Supplementary Table 1). Because of the above consideration, the value L1/2 must be placed in the two fields corresponding to A open / B shut and to A shut / B open. This argument also follows from the consideration that the channels are indistinguishable, and thus swapping the labels “A” and “B” must result in the same contingency table. Thus, the contingency table must be symmetric.

|  | A open | A shut | Marginal sum |
| --- | --- | --- | --- |
| B open | $L2$ | $L1/2$ | L2+L1/2 |
| B shut | $L1/2$ | $L0$ | L1/2+L0 |
| Marginal sum | L2+L1/2 | L1/2+L0 | L0+L1+L2=*n* |

**Supplementary Table 1**.

To calculate the respective fractions, we divide $L0$, $L1$ and $L2$ by the number of sweeps:

$$f_{0}=\frac{L0}{n}{, f}_{1}=\frac{L1}{n}, f_{2}=\frac{L2}{n}$$

The corresponding contingency table becomes Supplementary Table 2, in which, because the channels are identical, f_open_ and f_shut_ apply to both channels and represent the fractions of open and closed channels, respectively:

|  | A open | A shut | Marginal sum |
| --- | --- | --- | --- |
| B open | $f_{2}$ | $f_{1}/2$ | $f_{\mathrm{open}}$ |
| B shut | $f_{1}/2$ | $f_{0}$ | $f_{\mathrm{shut}}$ |
| Marginal sum | $f_{\mathrm{open}}$ | $f_{\mathrm{shut}}$ | 1 |

**Supplementary Table 2**.

Supplementary Table 2 corresponds to Eq. 7 in the main article. It pertains to identical indistinguishable channels, irrespective of whether the channels interact or not.

By knowing $f_{\mathrm{open}}$ and$f_{\mathrm{shut}}$ from the marginal sums in Supplementary Table 2, one can calculate the fractions $\bar{f_{0}}$, $\bar{f_{1}}$ and $\bar{f_{2}}$ that would be expected (as a null hypothesis) if the channels were independent (noninteracting) by multiplying the marginal sums by each other, as shown in Supplementary Table 3:

|  | A open | A shut | Marginal sum |
| --- | --- | --- | --- |
| B open | $\bar{f_{2}}=f_{\mathrm{open}}\cdot f_{\mathrm{open}}$ | $\bar{f_{1}}/2=f_{\mathrm{shut}}\cdot f_{\mathrm{open}}$ | $f_{\mathrm{open}}$ |
| B shut | $\bar{f_{1}}/2=f_{\mathrm{open}}\cdot f_{\mathrm{shut}}$ | $\bar{f_{0}}=f_{\mathrm{shut}}\cdot f_{\mathrm{shut}}$ | $f_{\mathrm{shut}}$ |
| Marginal sum | $f_{\mathrm{open}}$ | $f_{\mathrm{shut}}$ | 1 |

**Supplementary Table 3**.

Supplementary Table 3 corresponds to Eq. 8 in the main article. Note that by construction, the marginal sums are the same as in Supplementary Table 2.

Then, by multiplying these expected fractions by the number of sweeps *n*, we obtain the values $\bar{L0},\bar{L1}$ and $\bar{L2}$ that would be expected under the assumption (null hypothesis) that the channels are not interacting:

$$\bar{L0}=n \cdot\bar{f_{0}}, \bar{L1}=n \cdot\bar{f_{1}}, \bar{L2}=n \cdot\bar{f_{2}}.$$

The corresponding contingency table is Supplementary Table 4. Note that by construction, the marginal sums are the same as in Supplementary Table 1.

|  | A open | A shut | Marginal sum |
| --- | --- | --- | --- |
| B open | $\bar{L2}$ | $\bar{L1}/2$ | $\bar{L2}+\bar{L1}/2=$L2+L1/2 |
| B shut | $\bar{L1}/2$ | $\bar{L0}$ | $\bar{L1}/2+\bar{L0}$=L1/2+L0 |
| Marginal sum | $\bar{L2}+\bar{L1}/2=$L2+L1/2 | $\bar{L1}/2+\bar{L0}$=L1/2+L0 | $\bar{L0}+\bar{L1}+\bar{L2}$= L0+L1+L2 = *n* |

**Supplementary Table 4**.

By design, the χ^2^ test ascertains the significance of the difference between the contingency table of the observed counts (Supplementary Table 1) and the contingency table of the counts expected under the null hypothesis of independence (Supplementary Table 4) (Howell, 2011). Alternatively, Fisher’s exact test ascertains the probability (using hypergeometric distributions) that the contingency table of the number of observed events (Supplementary Table 1) deviates at least as much from the table under the null hypothesis of independence (Supplementary Table 4) (Sprent, 2011). Thus for identical indistinguishable channels, these two tests are suitable to assess the presence of interactions based on a triplet of counts {L0, L1, L2}.

*Numerical example*

As an example, we consider L0 = 68, L1 = 24 and L2 = 8, with *n* = 100.

The contingency table of the observed counts is shown as Supplementary Table 5, corresponding to Supplementary Table 1.

|  | A open | A shut | Marginal sum |
| --- | --- | --- | --- |
| B open | 8 | 12 | 20 |
| B shut | 12 | 68 | 80 |
| Marginal sum | 20 | 80 | 100 |

**Supplementary Table 5**.

The contingency table of the observed fractions is shown as Supplementary Table 6, corresponding to Supplementary Table 2.

|  | A open | A shut | Marginal sum |
| --- | --- | --- | --- |
| B open | 0.08 | 0.12 | $f_{\mathrm{open}}$ = 0.2 |
| B shut | 0.12 | 0.68 | $f_{\mathrm{shut}}$ = 0.8 |
| Marginal sum | $f_{\mathrm{open}}$ = 0.2 | $f_{\mathrm{shut}}$ = 0.8 | 1 |

**Supplementary Table 6**.

The contingency table of the fractions that would be expected from the marginal sums under the assumption of independence is shown as Supplementary Table 7, corresponding to Supplementary Table 3.

|  | A open | A shut | Marginal sum |
| --- | --- | --- | --- |
| B open | $\bar{f_{2}}=0.04$ | $\bar{f_{1}}/2=0.16$ | $f_{\mathrm{open}}$ |
| B shut | $\bar{f_{1}}/2=0.16$ | $\bar{f_{0}}=0.64$ | $f_{\mathrm{shut}}$ |
| Marginal sum | $f_{\mathrm{open}}$ | $f_{\mathrm{shut}}$ | 1 |

**Supplementary Table 7**.

From Supplementary Table 7, we have $\bar{f_{0}}$ = 0.64, $\bar{f_{1}}$ = 0.32, and $\bar{f_{2}}$ = 0.04.

The contingency table of the counts that would be expected from the marginal sums under the assumption of independence is shown as Supplementary Table 8, corresponding to Supplementary Table 4.

|  | A open | A shut | Marginal sum |
| --- | --- | --- | --- |
| B open | $\bar{L2}$ = 4 | $\bar{L1}/2$ = 16 | 20 |
| B shut | $\bar{L1}/2$ = 16 | $\bar{L0}$ = 64 | 80 |
| Marginal sum | 20 | 80 | 100 |

**Supplementary Table 8**.

From Supplementary Table 4, we obtain $\bar{L0}=64, \bar{L1}$ = 32, and $\bar{L2}$ = 4.

The p-value of the χ^2^ test applied on these data is 0.0124. The p-value of Fisher’s exact test is 0.0247. Thus, in this numerical example, the null hypothesis of independence (i.e., noninteracting channels) is rejected by both tests at the p<0.05 level.

*References*

Clatot, J., Hoshi, M., Wan, X., Liu, H., Jain, A., Shinlapawittayatorn, K., Marionneau, C., Ficker, E., Ha, T., and Deschenes, I. (2017). Voltage-gated sodium channels assemble and gate as dimers. *Nat Commun* 8, 2077.

Howell, D.C. (2011). "Chi-square test: analysis of contingency tables" in *International encyclopedia of statistical science,* ed. M. Lovric. (Berlin Heidelberg: Springer), 250-252.

Sprent, P. (2011). "Fisher exact test" in *International encyclopedia of statistical science,* ed. M. Lovric. (Berlin Heidelberg: Springer), 524-525.

**Illustration of the Kronecker sum for two 2-state C↔O Markovian models**

We consider the transition rate matrices for two different channels A and B:

$\mathbf{Q}_{A}=\left[ \begin{matrix} -\alpha_{a} & \beta_{a} \\ \alpha_{a} & -\beta_{a} \end{matrix} \right]$, $\mathbf{Q}_{B}=\left[ \begin{matrix} -\alpha_{b} & \beta_{b} \\ \alpha_{b} & -\beta_{b} \end{matrix} \right]$

The corresponding probability vectors are:

$\mathbf{p}_{A}=\left[ \begin{matrix} p_{A,closed} \\ p_{A,open} \end{matrix} \right]$ and $\mathbf{p}_{B}=\left[ \begin{matrix} p_{B,closed} \\ p_{B,open} \end{matrix} \right]$

The dynamics of the models is described as follows:

$\frac{d}{dt}\mathbf{p}_{A}=\mathbf{Q}_{A}\mathbf{p}_{A}$, and $\frac{d}{dt}\mathbf{p}_{B}=\mathbf{Q}_{B}\mathbf{p}_{B}$

In the following, $\otimes$ denotes the Kronecker product, $\oplus$ denotes the Kronecker sum.

The 2 x 2 identity matrix is:

$$\mathbf{I}_{A}= \mathbf{I}_{B}=\left[ \begin{matrix} 1 & 0 \\ 0 & 1 \end{matrix} \right]$$

The Kronecker sum of **Q**_A_ and **Q**_B_ is defined as

$$\mathbf{Q}_{\mathrm{AB}}={\mathbf{Q}_{A}\oplus\mathbf{Q}_{B}=\mathbf{Q}}_{A}\otimes\mathbf{I}_{\mathbf{B}}+\mathbf{I}_{\mathbf{A}}\otimes\mathbf{Q}_{B}$$

with

$$\mathbf{Q}_{A}\otimes\mathbf{I}_{\mathbf{B}}\boldsymbol{=}\left[ \begin{matrix} -\alpha_{a} & \beta_{a} \\ \alpha_{a} & -\beta_{a} \end{matrix} \right]\otimes\left[ \begin{matrix} 1 & 0 \\ 0 & 1 \end{matrix} \right]= \left[ \begin{matrix} -\alpha_{a}\left[ \begin{matrix} 1 & 0 \\ 0 & 1 \end{matrix} \right] & \beta_{a}\left[ \begin{matrix} 1 & 0 \\ 0 & 1 \end{matrix} \right] \\ \alpha_{a}\left[ \begin{matrix} 1 & 0 \\ 0 & 1 \end{matrix} \right] & -\beta_{a}\left[ \begin{matrix} 1 & 0 \\ 0 & 1 \end{matrix} \right] \end{matrix} \right]= \left[ \begin{aligned} \begin{matrix} -\alpha_{a} & 0 \\ 0 & -\alpha_{a} \end{matrix} \begin{matrix} \beta_{a} & 0 \\ 0 & \beta_{a} \end{matrix} \\ \begin{matrix} \alpha_{a} & 0 \\ 0 & \alpha_{a} \end{matrix} \begin{matrix} -\beta_{a} & 0 \\ 0 & -\beta_{a} \end{matrix} \end{aligned} \right]$$

$$\mathbf{I}_{\mathbf{A}}\otimes\mathbf{Q}_{B}\boldsymbol{=}\left[ \begin{matrix} 1 & 0 \\ 0 & 1 \end{matrix} \right] \otimes\left[ \begin{matrix} -\alpha_{b} & \beta_{b} \\ \alpha_{b} & -\beta_{b} \end{matrix} \right]=\left[ \begin{matrix} 1\left[ \begin{matrix} -\alpha_{b} & \beta_{b} \\ \alpha_{b} & -\beta_{b} \end{matrix} \right] & 0\left[ \begin{matrix} -\alpha_{b} & \beta_{b} \\ \alpha_{b} & -\beta_{b} \end{matrix} \right] \\ 0\left[ \begin{matrix} -\alpha_{b} & \beta_{b} \\ \alpha_{b} & -\beta_{b} \end{matrix} \right] & 1\left[ \begin{matrix} -\alpha_{b} & \beta_{b} \\ \alpha_{b} & -\beta_{b} \end{matrix} \right] \end{matrix} \right]=\left[ \begin{aligned} \begin{matrix} -\alpha_{b} & \beta_{b} \\ \alpha_{b} & -\beta_{b} \end{matrix} \begin{matrix} 0 & 0 \\ 0 & 0 \end{matrix} \\ \begin{matrix} 0 & 0 \\ 0 & 0 \end{matrix} \begin{matrix} -\alpha_{b} & \beta_{b} \\ \alpha_{b} & -\beta_{b} \end{matrix} \end{aligned} \right]$$

and thus,

$$\mathbf{Q}_{\mathrm{AB}}\boldsymbol{=}\left[ \begin{aligned} \begin{matrix} -\alpha_{a} & 0 \\ 0 & -\alpha_{a} \end{matrix} \begin{matrix} \beta_{a} & 0 \\ 0 & \beta_{a} \end{matrix} \\ \begin{matrix} \alpha_{a} & 0 \\ 0 & \alpha_{a} \end{matrix} \begin{matrix} -\beta_{a} & 0 \\ 0 & -\beta_{a} \end{matrix} \end{aligned} \right]+\left[ \begin{aligned} \begin{matrix} -\alpha_{b} & \beta_{b} \\ \alpha_{b} & -\beta_{b} \end{matrix} \begin{matrix} 0 & 0 \\ 0 & 0 \end{matrix} \\ \begin{matrix} 0 & 0 \\ 0 & 0 \end{matrix} \begin{matrix} -\alpha_{b} & \beta_{b} \\ \alpha_{b} & -\beta_{b} \end{matrix} \end{aligned} \right]=\left[ \begin{matrix} \begin{matrix} -\alpha_{a}-\alpha_{b} & \beta_{b} \\ \alpha_{b} & -\alpha_{a}-\beta_{b} \end{matrix} & \begin{matrix} \beta_{a} & 0 \\ 0 & \beta_{a} \end{matrix} \\ \begin{matrix} \alpha_{a} & 0 \\ 0 & \alpha_{a} \end{matrix} & \begin{matrix} -\beta_{a}-\alpha_{b} & \beta_{b} \\ \alpha_{b} & -\beta_{a}-\beta_{b} \end{matrix} \end{matrix} \right]$$

Note that the sum of every column is 0.

The corresponding vector of probabilities is:

$$\mathbf{p}_{\mathrm{AB}}=\left[ \begin{matrix} \begin{matrix} p_{A,closed;B,closed} \\ p_{A,closed;B,open} \end{matrix} \\ \begin{matrix} p_{A,open;B,closed} \\ p_{A,open;B,open} \end{matrix} \end{matrix} \right]$$

The dynamic of the channel pair is then described by:

$$\frac{d}{dt}\mathbf{p}_{\mathrm{AB}}=\mathbf{Q}_{\mathrm{AB}}\mathbf{p}_{\mathrm{AB}}$$

i.e.,

$\frac{d}{dt}\left[ \begin{matrix} \begin{matrix} p_{A,closed;B,closed} \\ p_{A,closed;B,open} \end{matrix} \\ \begin{matrix} p_{A,open;B,closed} \\ p_{A,open;B,open} \end{matrix} \end{matrix} \right]=\left[ \begin{matrix} \begin{matrix} -\alpha_{a}-\alpha_{b} & \beta_{b} \\ \alpha_{b} & -\alpha_{a}-\beta_{b} \end{matrix} & \begin{matrix} \beta_{a} & 0 \\ 0 & \beta_{a} \end{matrix} \\ \begin{matrix} \alpha_{a} & 0 \\ 0 & \alpha_{a} \end{matrix} & \begin{matrix} -\beta_{a}-\alpha_{b} & \beta_{b} \\ \alpha_{b} & -\beta_{a}-\beta_{b} \end{matrix} \end{matrix} \right]\cdot\left[ \begin{matrix} \begin{matrix} p_{A,closed;B,closed} \\ p_{A,closed;B,open} \end{matrix} \\ \begin{matrix} p_{A,open;B,closed} \\ p_{A,open;B,open} \end{matrix} \end{matrix} \right]$.

For two identical channels, we have:

$$\mathbf{Q}_{A}=\mathbf{Q}_{B}=\left[ \begin{matrix} -\alpha& \beta\\ \alpha& -\beta\end{matrix} \right]$$

and

$\mathbf{Q}_{\mathrm{AB}}=\left[ \begin{matrix} \begin{matrix} -2\alpha& \beta\\ \alpha& -\alpha-\beta\end{matrix} & \begin{matrix} \beta& 0 \\ 0 & \beta\end{matrix} \\ \begin{matrix} \alpha& 0 \\ 0 & \alpha\end{matrix} & \begin{matrix} -\beta-\alpha& \beta\\ \alpha& -2\beta\end{matrix} \end{matrix} \right]$,

with

$\frac{d}{dt}\left[ \begin{matrix} \begin{matrix} p_{A,closed;B,closed} \\ p_{A,closed;B,open} \end{matrix} \\ \begin{matrix} p_{A,open;B,closed} \\ p_{A,open;B,open} \end{matrix} \end{matrix} \right]=\left[ \begin{matrix} \begin{matrix} -2\alpha& \beta\\ \alpha& -\alpha-\beta\end{matrix} & \begin{matrix} \beta& 0 \\ 0 & \beta\end{matrix} \\ \begin{matrix} \alpha& 0 \\ 0 & \alpha\end{matrix} & \begin{matrix} -\beta-\alpha& \beta\\ \alpha& -2\beta\end{matrix} \end{matrix} \right]\cdot\left[ \begin{matrix} \begin{matrix} p_{A,closed;B,closed} \\ p_{A,closed;B,open} \end{matrix} \\ \begin{matrix} p_{A,open;B,closed} \\ p_{A,open;B,open} \end{matrix} \end{matrix} \right]$.

**Operators O_A_ and O_B_ to extract the state occupancy probability vectors p_A_ and p_B_ from p_AB_: example for a composite model of two 2-state channels**

We consider the following composite model of two 2-state channels A and B (see previous section), with the following transition rate matrix $\mathbf{Q}_{\mathrm{AB}}$:

$$\mathbf{Q}_{\mathrm{AB}}\boldsymbol{=}\left[ \begin{matrix} \begin{matrix} -\alpha_{a}-\alpha_{b} & \beta_{b} \\ \alpha_{b} & -\alpha_{a}-\beta_{b} \end{matrix} & \begin{matrix} \beta_{a} & 0 \\ 0 & \beta_{a} \end{matrix} \\ \begin{matrix} \alpha_{a} & 0 \\ 0 & \alpha_{a} \end{matrix} & \begin{matrix} -\beta_{a}-\alpha_{b} & \beta_{b} \\ \alpha_{b} & -\beta_{a}-\beta_{b} \end{matrix} \end{matrix} \right]$$

The corresponding probability vector is:

$$\mathbf{p}_{\mathrm{AB}}=\left[ \begin{matrix} \begin{matrix} p_{A,closed;B,closed} \\ p_{A,closed;B,open} \end{matrix} \\ \begin{matrix} p_{A,open;B,closed} \\ p_{A,open;B,open} \end{matrix} \end{matrix} \right]$$

The operators **O**_A_ and **O**_B_ are constructed as

$$\mathbf{O}_{A}=\mathbf{I}_{A}\otimes\mathbf{1}_{A}^{T}=\left[ \begin{matrix} 1 & 0 \\ 0 & 1 \end{matrix} \right]\otimes\left[ 1 1 \right]=\left[ \begin{matrix} 1 \left[ 1 1 \right] & 0 \left[ 1 1 \right] \\ 0 \left[ 1 1 \right] & 1 \left[ 1 1 \right] \end{matrix} \right]=\left[ \begin{matrix} 1 & 1 \\ 0 & 0 \end{matrix} \begin{matrix} 0 & 0 \\ 1 & 1 \end{matrix} \right]$$

$$\mathbf{O}_{B}=\mathbf{1}_{A}^{T}\otimes\mathbf{I}_{B}=\left[ 1 1 \right]\otimes\left[ \begin{matrix} 1 & 0 \\ 0 & 1 \end{matrix} \right]=\left[ 1\left[ \begin{matrix} 1 & 0 \\ 0 & 1 \end{matrix} \right] 1\left[ \begin{matrix} 1 & 0 \\ 0 & 1 \end{matrix} \right] \right]=\left[ \begin{matrix} 1 & 0 \\ 0 & 1 \end{matrix} \begin{matrix} 1 & 0 \\ 0 & 1 \end{matrix} \right]$$

**O**_A_ and **O**_B_ permit to extract **p**_A_ and **p**_B_ from **p**_AB_ as follows:

$\mathbf{p}_{A}=\mathbf{O}_{A}\mathbf{p}_{\mathrm{AB}}$

$\mathbf{p}_{A}$ $=\left[ \begin{matrix} 1 & 1 \\ 0 & 0 \end{matrix} \begin{matrix} 0 & 0 \\ 1 & 1 \end{matrix} \right]\cdot\left[ \begin{matrix} \begin{matrix} p_{A,closed;B,closed} \\ p_{A,closed;B,open} \end{matrix} \\ \begin{matrix} p_{A,open;B,closed} \\ p_{A,open;B,open} \end{matrix} \end{matrix} \right]=\left[ \begin{matrix} p_{A,closed;B,closed}+p_{A,closed;B,open} \\ p_{A,open;B,closed}+p_{A,open;B,open} \end{matrix} \right]=\left[ \begin{matrix} p_{A,closed} \\ p_{A,open} \end{matrix} \right]$

$$\mathbf{p}_{B}=\mathbf{O}_{B}\mathbf{p}_{\mathrm{AB}}$$

$\mathbf{p}_{B}=\left[ \begin{matrix} 1 & 0 \\ 0 & 1 \end{matrix} \begin{matrix} 1 & 0 \\ 0 & 1 \end{matrix} \right]\cdot\left[ \begin{matrix} \begin{matrix} p_{A,closed;B,closed} \\ p_{A,closed;B,open} \end{matrix} \\ \begin{matrix} p_{A,open;B,closed} \\ p_{A,open;B,open} \end{matrix} \end{matrix} \right]=\left[ \begin{matrix} p_{A,closed;B,closed}+p_{A,open;B,closed} \\ p_{A,closed;B,open}+p_{A,open;B,open} \end{matrix} \right]=\left[ \begin{matrix} p_{B,closed} \\ p_{B,open} \end{matrix} \right]$.

**Microscopic reversibility in composite models**

Here, we outline a proof based on Cartesian graph products (denoted by the symbol “□”) that the composite model of two Markovian models preserves microscopic reversibility if the original models do so. The proof is based on Kolmogorov’s criterion, stating that for every loop in the Markovian model, the product of the transition rates in one direction along the loop must be equal to the product of the transition rates in the reverse direction.

The proof consists in considering all possible subgraphs of the composite model arising as the Cartesian graph product of subgraphs of the original models.

We consider first the product of two edges (involving two states), as illustrated in the following figure:


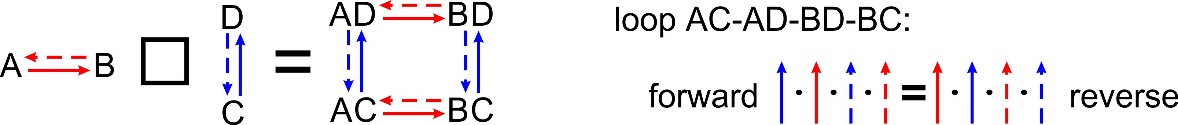


Here, A↔B and C↔D can refer to any edges in the original models (e.g., C3↔C2 and IF↔IS in the Clancy-Rudy model). As illustrated graphically, the product of the rates (depicted by arrows of different colors and styles) in the forward and reverse direction in the AC-AD-BD-BC loop of the composite model are equal, by virtue of the commutativity of multiplication. Thus, the resulting subgraph satisfies Kolmogorov’s criterion.

We consider now the product of one edge and one loop, as illustrated below:


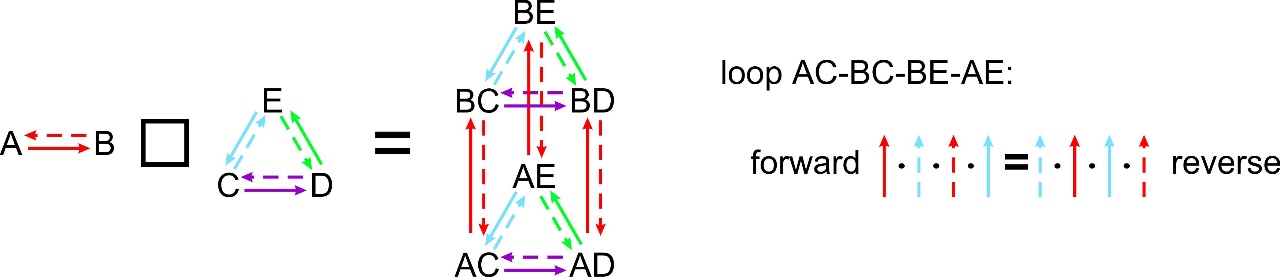


The resulting subgraph contains 2 triangular loops and 3 loops involving 4 composite states. The two triangular loops AC-AD-AE and BC-BD-BE consist of the same arrangement of rates as the original subgraph C-D-E. Thus, if Kolmogorov’s criterion is satisfied in the original C-D-E loop, it is also satisfied in the two triangular loops of the composite subgraph. Regarding the remaining loops involving 4 states, the product of the rates in the forward and reverse directions are always equal, as illustrated graphically for the AC-AD-BD-BC loop of the composite model. Thus, Kolmogorov’s criterion is satisfied for all loops in the subgraph of the composite model.

Next, we consider the product of two triangular loops (such as the C1-O-IF loop in the Clancy-Rudy model), as illustrated below:


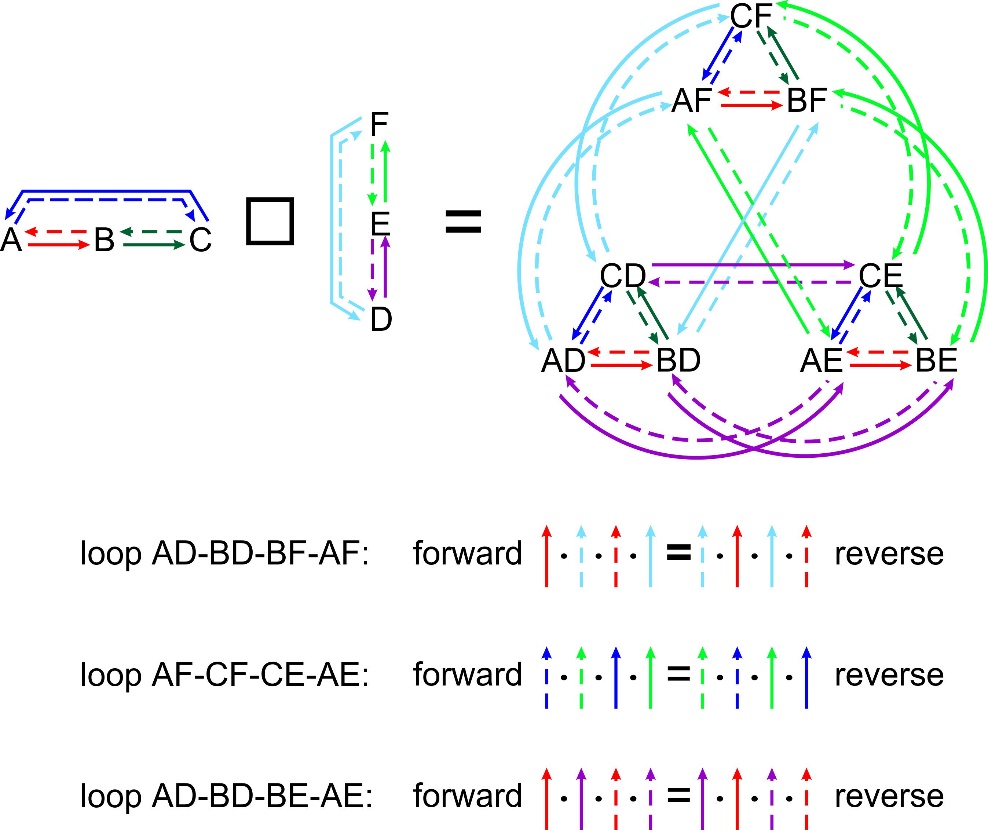


The resulting subgraph (redrawn here for clarity with a different layout but without changing its connectivity) contains 6 triangular loops and 12 loops involving 4 composite states. All the six triangular loops (such as AD-BD-CD or AD-AE-AF) consist of the same arrangement of rates as the original subgraphs A-B-C or D-E-F. Thus, if Kolmogorov’s criterion is satisfied in the original A-B-C and D-E-F loops, it is also satisfied in the 6 triangular loops of the composite subgraph. Regarding the remaining loops involving 4 states, the product of the rates in the forward and reverse directions are again always equal, as exemplified graphically for three different loops in the composite model. Thus, Kolmogorov’s criterion is again satisfied for all loops in the subgraph of the composite model.

These considerations pertain to any subgraph of the composite model arising as the Cartesian product of subgraphs from the original models, and can be extended to loops with 4, 5 or more states. Since Kolmogorov’s criterion is satisfied for all possible composite model subgraphs if it is satisfied in the original model graphs, we conclude that the composite model of two Markovian models preserves microscopic reversibility if the original models do so.

**Example operations of changing the free energies of states and of barriers between states**

We consider the following composite model of two 2-state channels A and B (see previous sections), with the following transition rate matrix $\mathbf{Q}_{\mathrm{AB}}$:

$$\mathbf{Q}_{\mathrm{AB}}\boldsymbol{=}\left[ \begin{matrix} \begin{matrix} -\alpha_{a}-\alpha_{b} & \beta_{b} \\ \alpha_{b} & -\alpha_{a}-\beta_{b} \end{matrix} & \begin{matrix} \beta_{a} & 0 \\ 0 & \beta_{a} \end{matrix} \\ \begin{matrix} \alpha_{a} & 0 \\ 0 & \alpha_{a} \end{matrix} & \begin{matrix} -\alpha_{b}-\beta_{a} & \beta_{b} \\ \alpha_{b} & -\beta_{a}-\beta_{b} \end{matrix} \end{matrix} \right]$$

The corresponding probability vector is:

$$\mathbf{p}_{\mathrm{AB}}=\left[ \begin{matrix} \begin{matrix} p_{A,closed;B,closed} \\ p_{A,closed;B,open} \end{matrix} \\ \begin{matrix} p_{A,open;B,closed} \\ p_{A,open;B,open} \end{matrix} \end{matrix} \right]$$

The equation governing the dynamics of **p**_AB_ is:

$$\frac{d}{dt}\mathbf{p}_{\mathrm{AB}}=\mathbf{Q}_{\mathrm{AB}}\mathbf{p}_{\mathrm{AB}}$$

i.e.,

$\frac{d}{dt}\left[ \begin{matrix} \begin{matrix} p_{A,closed;B,closed} \\ p_{A,closed;B,open} \end{matrix} \\ \begin{matrix} p_{A,open;B,closed} \\ p_{A,open;B,open} \end{matrix} \end{matrix} \right]=\left[ \begin{matrix} \begin{matrix} -\alpha_{a}-\alpha_{b} & \beta_{b} \\ \alpha_{b} & -\alpha_{a}-\beta_{b} \end{matrix} & \begin{matrix} \beta_{a} & 0 \\ 0 & \beta_{a} \end{matrix} \\ \begin{matrix} \alpha_{a} & 0 \\ 0 & \alpha_{a} \end{matrix} & \begin{matrix} -\alpha_{b}-\beta_{a} & \beta_{b} \\ \alpha_{b} & -\beta_{a}-\beta_{b} \end{matrix} \end{matrix} \right]\cdot\left[ \begin{matrix} \begin{matrix} p_{A,closed;B,closed} \\ p_{A,closed;B,open} \end{matrix} \\ \begin{matrix} p_{A,open;B,closed} \\ p_{A,open;B,open} \end{matrix} \end{matrix} \right]$.

*Elementary operations (raising the energy of a composite state or the energy barrier between composite states)*

In the original model, raising the energy of the composite state OO by 1 kT, with $\varepsilon=e^{E/kT}=e^{1}=e$, corresponds to multiplying the fourth column of **Q**_AB_ by ε:

$$\mathbf{Q}_{AB_{OO,raised by 1 kT}}\boldsymbol{=}\left[ \begin{matrix} \begin{matrix} -\alpha_{a}-\alpha_{b} & \beta_{b} \\ \alpha_{b} & -\alpha_{a}-\beta_{b} \end{matrix} & \begin{matrix} \beta_{a} & 0 \\ 0 & {\varepsilon\beta}_{a} \end{matrix} \\ \begin{matrix} \alpha_{a} & 0 \\ 0 & \alpha_{a} \end{matrix} & \begin{matrix} -\alpha_{b}-\beta_{a} & {\varepsilon\beta}_{b} \\ \alpha_{b} & \varepsilon(-\beta_{a}-\beta_{b}) \end{matrix} \end{matrix} \right]$$

In the original model, raising the energy of the two composite states CO/OC by 1 kT, with $\varepsilon=e^{E/kT}=e^{1}=e$ corresponds to multiplying the second and the third columns of **Q**_AB_ by ε:

$$\mathbf{Q}_{\mathrm{AB}_{CO/OC raised by 1 kT}}\boldsymbol{=}\left[ \begin{matrix} \begin{matrix} -\alpha_{a}-\alpha_{b} & {\varepsilon\beta}_{b} \\ \alpha_{b} & \varepsilon(-\alpha_{a}-\beta_{b}) \end{matrix} & \begin{matrix} {\varepsilon\beta}_{a} & 0 \\ 0 & \beta_{a} \end{matrix} \\ \begin{matrix} \alpha_{a} & 0 \\ 0 & \varepsilon\alpha_{a} \end{matrix} & \begin{matrix} \varepsilon(-\alpha_{b}-\beta_{a}) & \beta_{b} \\ {\varepsilon\alpha}_{b} & -\beta_{a}-\beta_{b} \end{matrix} \end{matrix} \right]$$

In the original model, raising the energy of the barriers CO-OO/OC-OO by 1 kT with $\sigma=e^{-E/kt}=e^{-1}$ corresponds to scaling the two transition rates between the two states separated by this barrier by$\sigma$. In the matrix **Q**_AB_, this corresponds to scaling by $\sigma$ the second and third elements of the fourth row and the second and third elements of the fourth column.

$$\mathbf{Q}_{\mathrm{AB}_{CO-OO/OC-OO raised by 1 kT}}\boldsymbol{=}\left[ \begin{matrix} \begin{matrix} -\alpha_{a}-\alpha_{b} & \beta_{b} \\ \alpha_{b} & -{\sigma\alpha}_{a}-\beta_{b} \end{matrix} & \begin{matrix} \beta_{a} & 0 \\ 0 & {\sigma\beta}_{a} \end{matrix} \\ \begin{matrix} \alpha_{a} & 0 \\ 0 & {\sigma\alpha}_{a} \end{matrix} & \begin{matrix} -{\sigma\alpha}_{b}-\beta_{a} & {\sigma\beta}_{b} \\ \sigma\alpha_{b} & \sigma(-\beta_{a}-\beta_{b}) \end{matrix} \end{matrix} \right]$$

Note that the diagonal elements must then be adjusted such that the sum of each column remains 0.

*Combinations of elementary operations*

In the $\mathbf{Q}_{\mathrm{AB}_{CO/OC raised by 1 kT}}$ model, raising the energy of the barriers CO-OO/OC-OO by 1 kT with $\sigma=e^{-E/kt}=e^{-1}$ corresponds to scaling the two transition rates between the two states separated by this barrier by$\sigma$:

$\mathbf{Q}_{\mathrm{AB}_{CO/OC raised by 1 kT; CO-OO/OC-OO raised by 1 kT}}$

$$\boldsymbol{=}\left[ \begin{matrix} \begin{matrix} -\alpha_{a}-\alpha_{b} & {\varepsilon\beta}_{b} \\ \alpha_{b} & -\sigma\varepsilon\alpha_{a}-{\varepsilon\beta}_{b} \end{matrix} & \begin{matrix} {\varepsilon\beta}_{a} & 0 \\ 0 & {\sigma\beta}_{a} \end{matrix} \\ \begin{matrix} \alpha_{a} & 0 \\ 0 & \varepsilon\sigma\alpha_{a} \end{matrix} & \begin{matrix} -\sigma\varepsilon\alpha_{b}-{\varepsilon\beta}_{a} & \sigma\beta_{b} \\ {\sigma\varepsilon\alpha}_{b} & \sigma(-\beta_{a}-\beta_{b}) \end{matrix} \end{matrix} \right]$$

In the $\mathbf{Q}_{\mathrm{AB}_{CO-OO/OC-OO raised by 1 kT}}$ model, raising the energy of the two composite states CO/OC by 1 kT, with $\varepsilon=e^{E/kT}=e^{1}=e$ corresponds to multiplying the second and the third columns of $\mathbf{Q}_{\mathrm{AB}_{CO-OO/OC-OO raised by 1 kT}}$ by ε:

$$\mathbf{Q}_{\mathrm{AB}_{CO-OO/OC-OO raised by 1 kT; CO/OC raised by 1 kT;}}$$

$$\boldsymbol{=}\left[ \begin{matrix} \begin{matrix} -\alpha_{a}-\alpha_{b} & {\varepsilon\beta}_{b} \\ \alpha_{b} & -\sigma\varepsilon\alpha_{a}-{\varepsilon\beta}_{b} \end{matrix} & \begin{matrix} {\varepsilon\beta}_{a} & 0 \\ 0 & {\sigma\beta}_{a} \end{matrix} \\ \begin{matrix} \alpha_{a} & 0 \\ 0 & \varepsilon\sigma\alpha_{a} \end{matrix} & \begin{matrix} -\sigma\varepsilon\alpha_{b}-{\varepsilon\beta}_{a} & \sigma\beta_{b} \\ {\sigma\varepsilon\alpha}_{b} & \sigma(-\beta_{a}-\beta_{b}) \end{matrix} \end{matrix} \right]$$

It can be noted that

$\mathbf{Q}_{\mathrm{AB}_{CO/OC raised by 1 kT; CO-OO/OC-OO raised by 1 kT}}\boldsymbol{=}\mathbf{Q}_{\mathrm{AB}_{CO-OO/OC-OO raised by 1 kT; O/OC raised by 1 kT;}}$,

which illustrates that these operations commute. This commutative property results from the commutativity of scalar multiplication and from the fact that each elementary operation corresponds to multiplying a given subset of elements by a given factor. The diagonal elements can either be recomputed after every elementary operation or only once at the end.


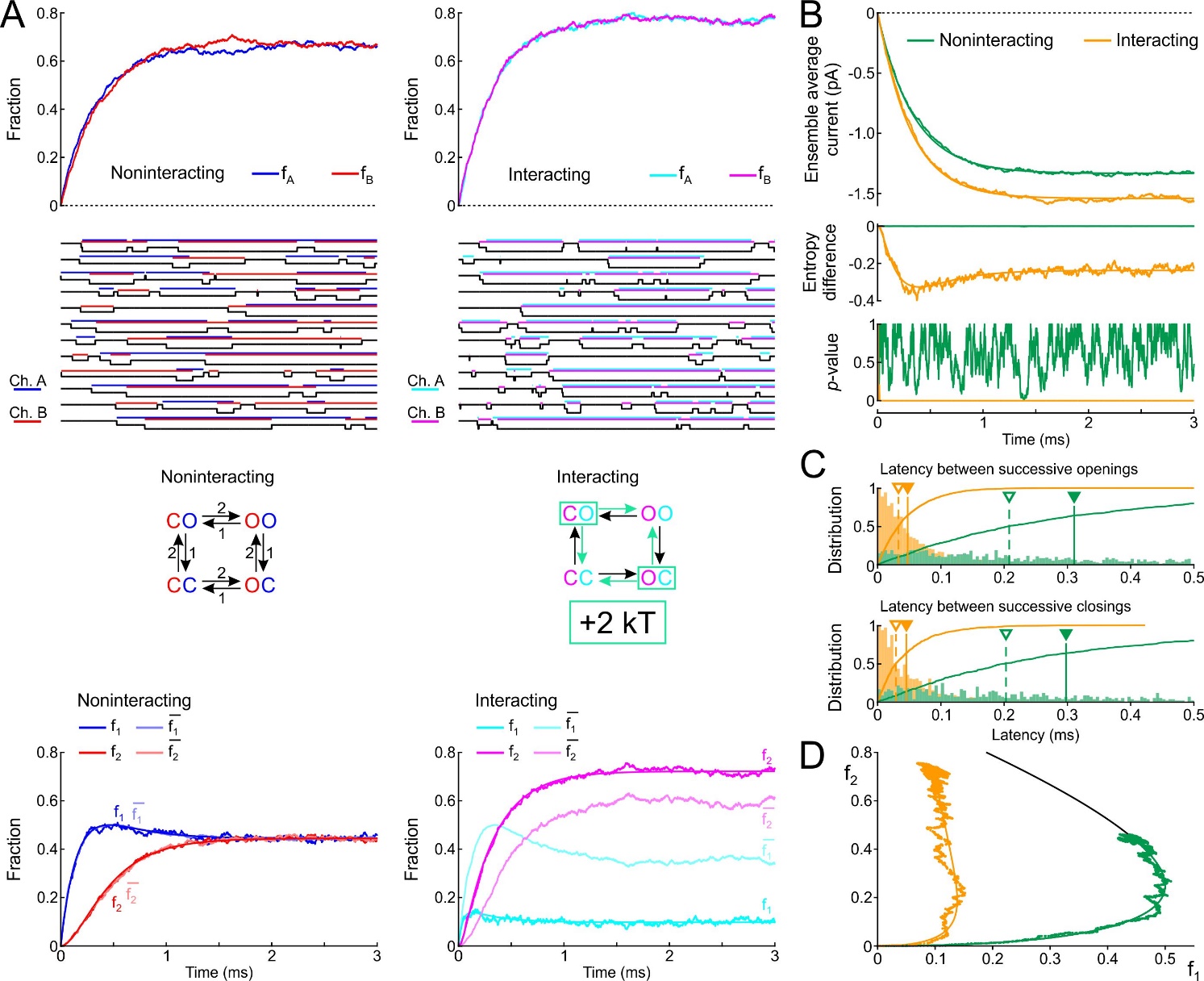


**Supplementary Figure S1**. Simulated gating behavior of a pair of 2-state channels (C: closed ↔ O: open; opening rate: 2 ms^−1^; closing rate: 1 ms^−1^) in the absence of interaction and upon raising the energy of the composite CO and OC states by 2 kT. Same protocol, analysis, and layout as in Figure 3 of the main article.


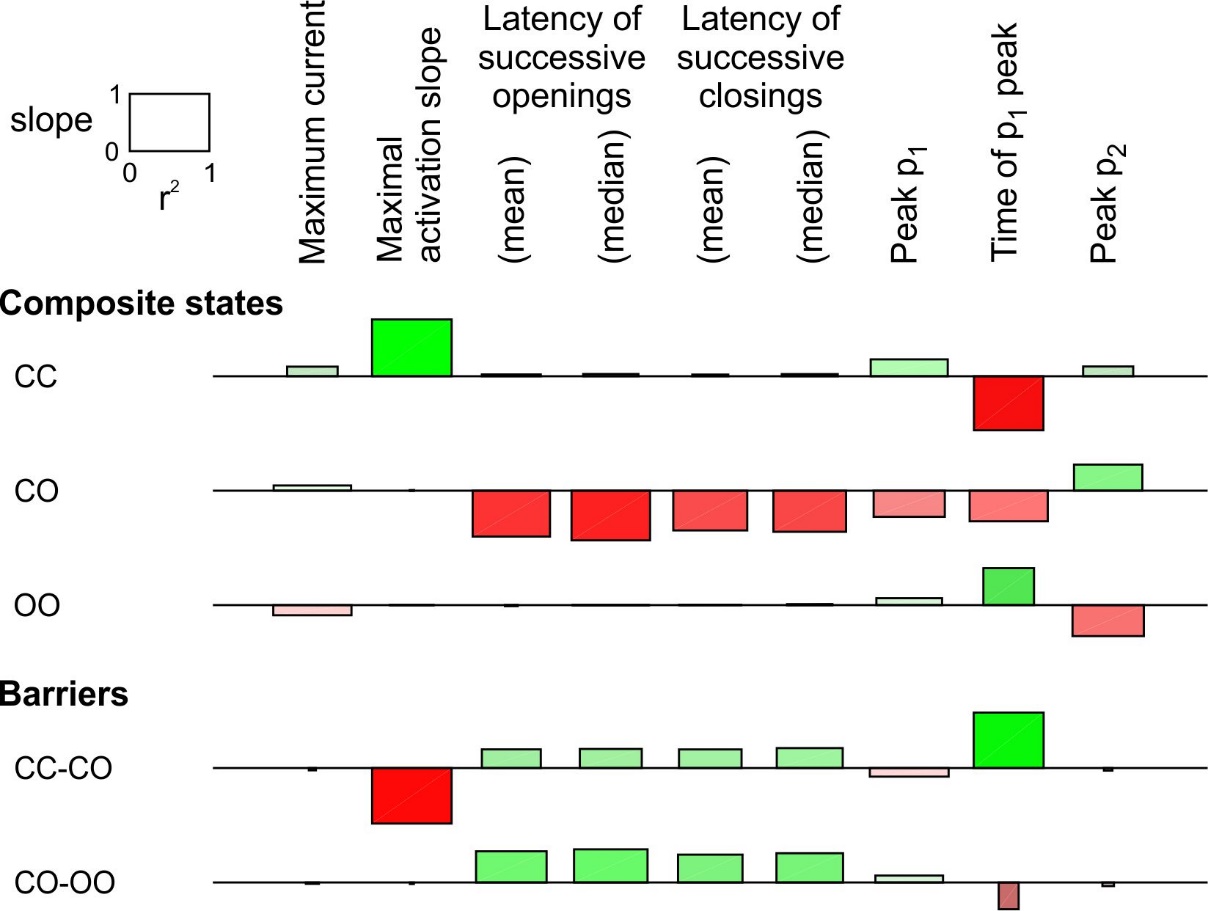


**Supplementary Figure S2.** Sensitivity analysis for the pair of 2-state channels (C: closed ↔ O: open; opening rate: 2 ms^−1^; closing rate: 1 ms^−1^). Same protocol, analysis, and layout as in Figure 4 of the main article.


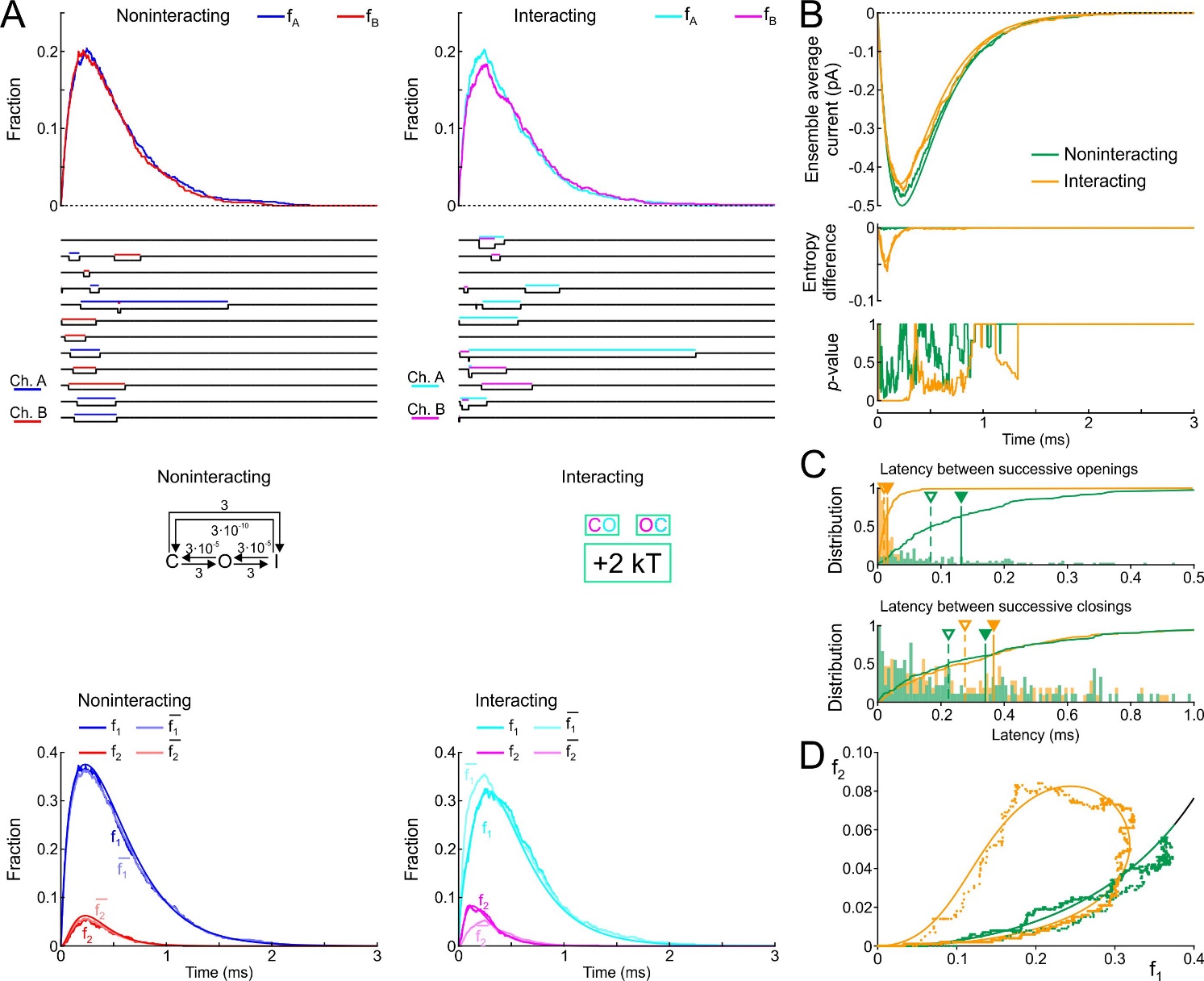


**Supplementary Figure S3.** Simulated gating behavior of a pair of 3-state channels (C: closed, O: open; and I: inactivated) using a triangular COI channel model in the absence of interaction and upon raising the energy of the composite CO and OC states by 2 kT. Same protocol, analysis, and layout as in Figures 3 and 5 of the main article. The rate constants of the triangular COI channel model are given in the directed graph in panel A.


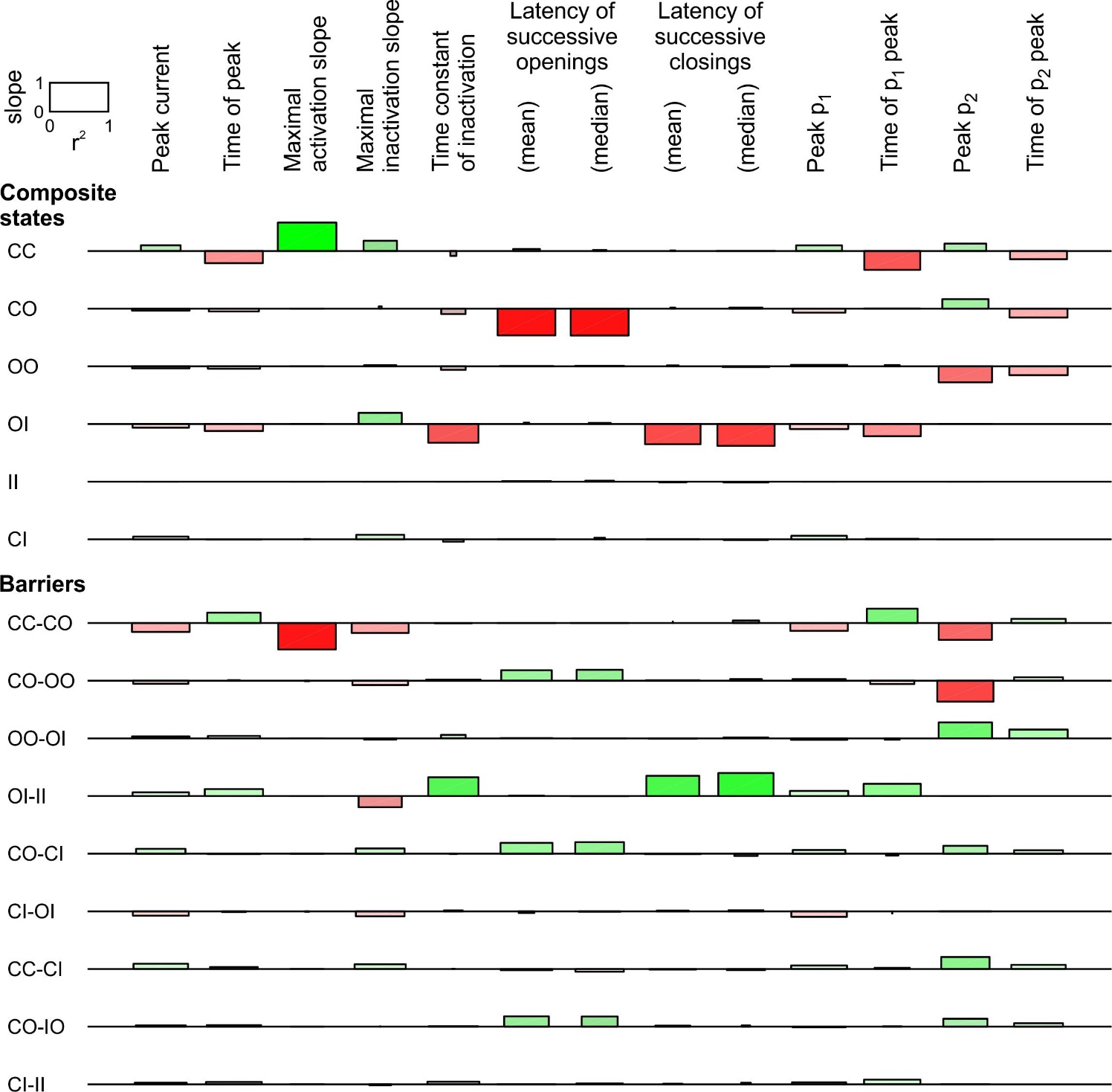


**Supplementary Figure S4.** Sensitivity analysis for the pair of 3-state channels represented each by the triangular COI model of Figure S3. Same protocol, analysis, and layout as in Figure 4 of the main article and Figure S2.


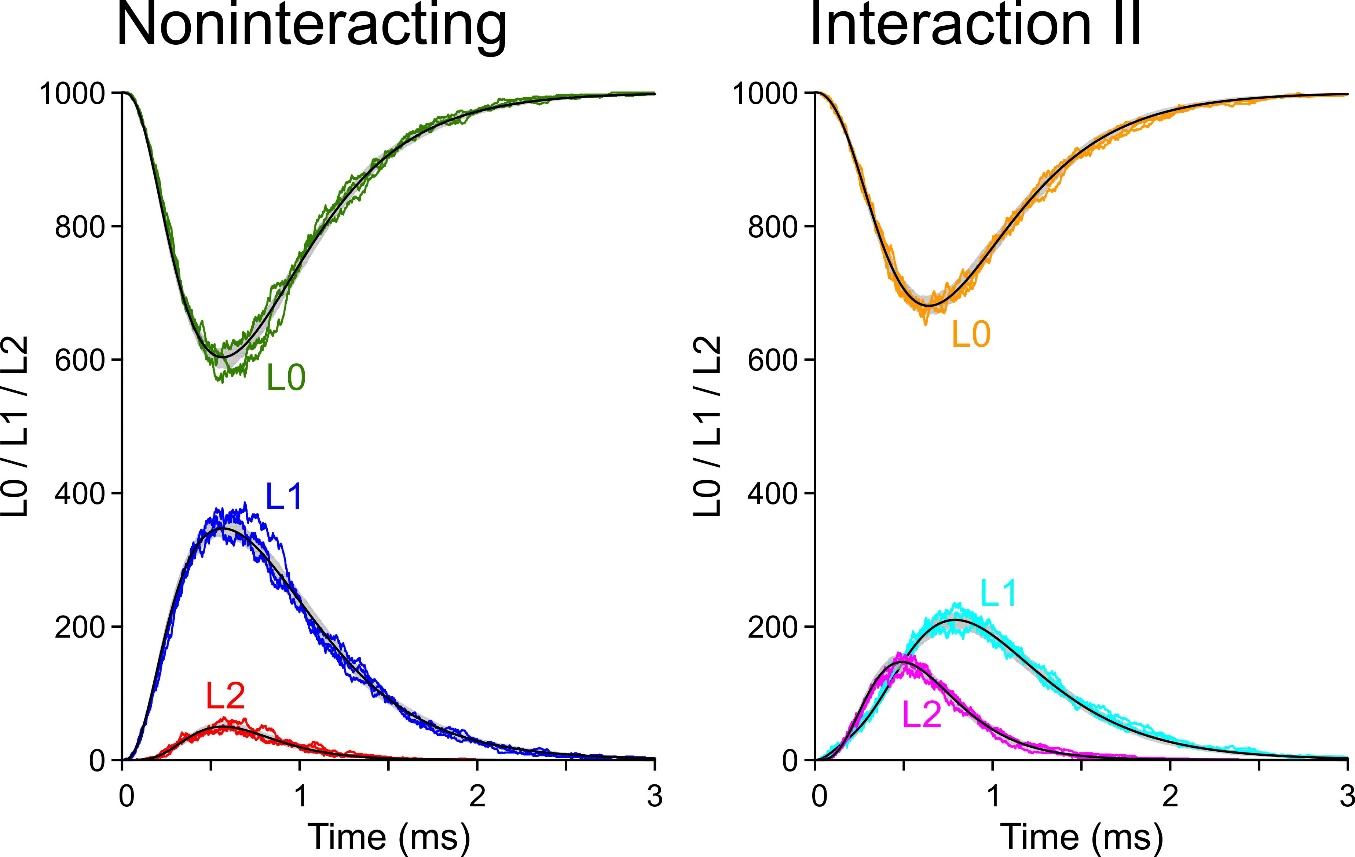


**Supplementary Figure S5.** The simulation of *n*=1000 sweeps of the wild-type Clancy-Rudy model pair without interaction (*left*) and with Interaction II (*right*, see Figure 9 of the main article) was repeated *m*=100 times to ascertain that L0, L1 and L2 counts converge, on average, to the values expected from deterministic simulations. The colored traces show 3 individual repeats, and the grey bands represent the mean ± standard deviation of the L0, L1 and L2 counts for all the 100 repeats. The values expected from deterministic simulations (*n*∙p_0_, *n*∙p_1_ and *n*∙p_2_) are represented by black curves. The figure shows that mean L0, L1 and L2 values closely correspond to the expectations *n*∙p_0_, *n*∙p_1_ and *n*∙p_2_.


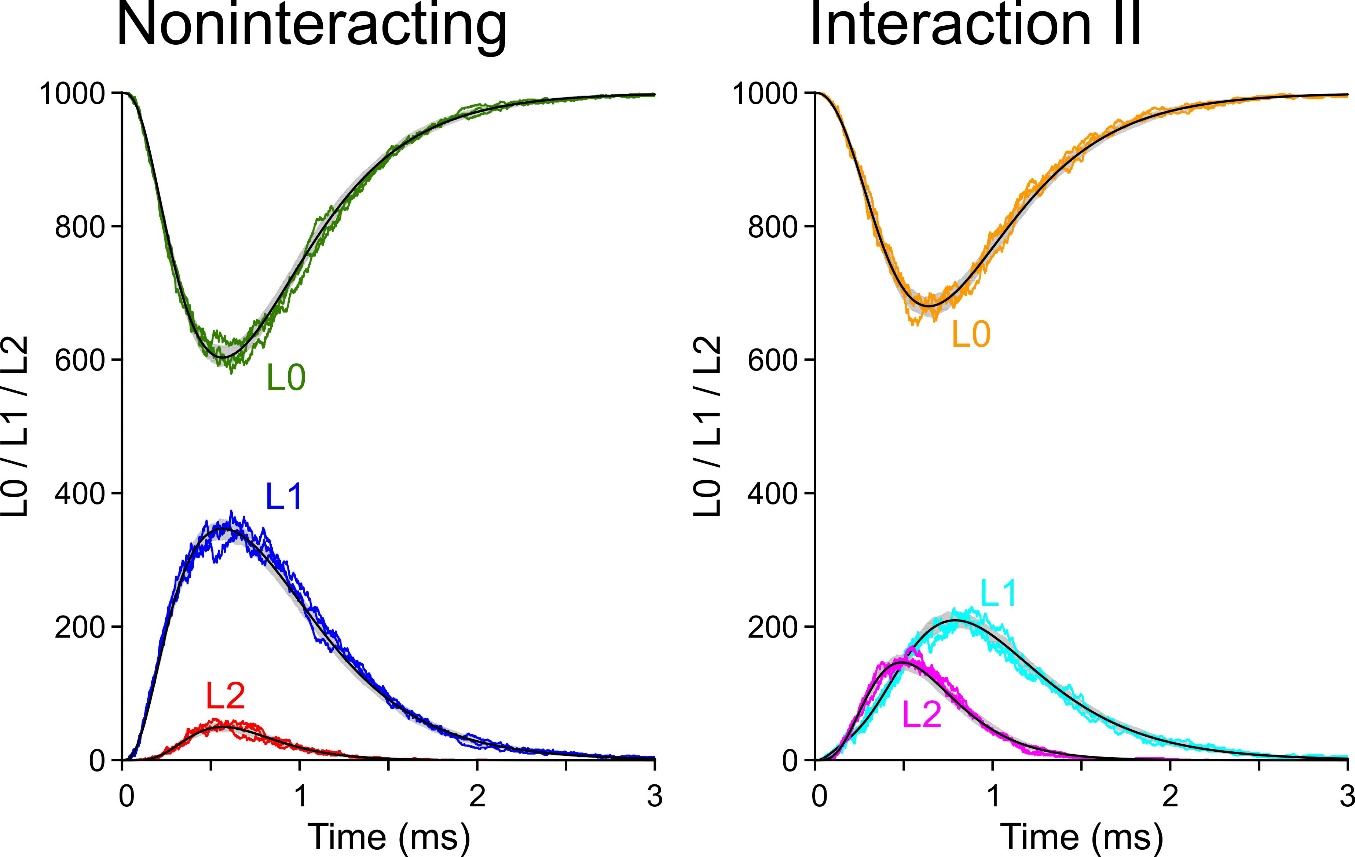


**Supplementary Figure S6.** The same simulations were conducted as in Supplementary Figure S5, but using Gillespie’s algorithm. Same layout as Supplementary Figure S5. The results are essentially the same as to those in Supplementary Figure S5 and show that mean L0, L1 and L2 values closely correspond to the expectations *n*∙p_0_, *n*∙p_1_ and *n*∙p_2_. These results validate our stochastic simulations based on matrix exponentials.


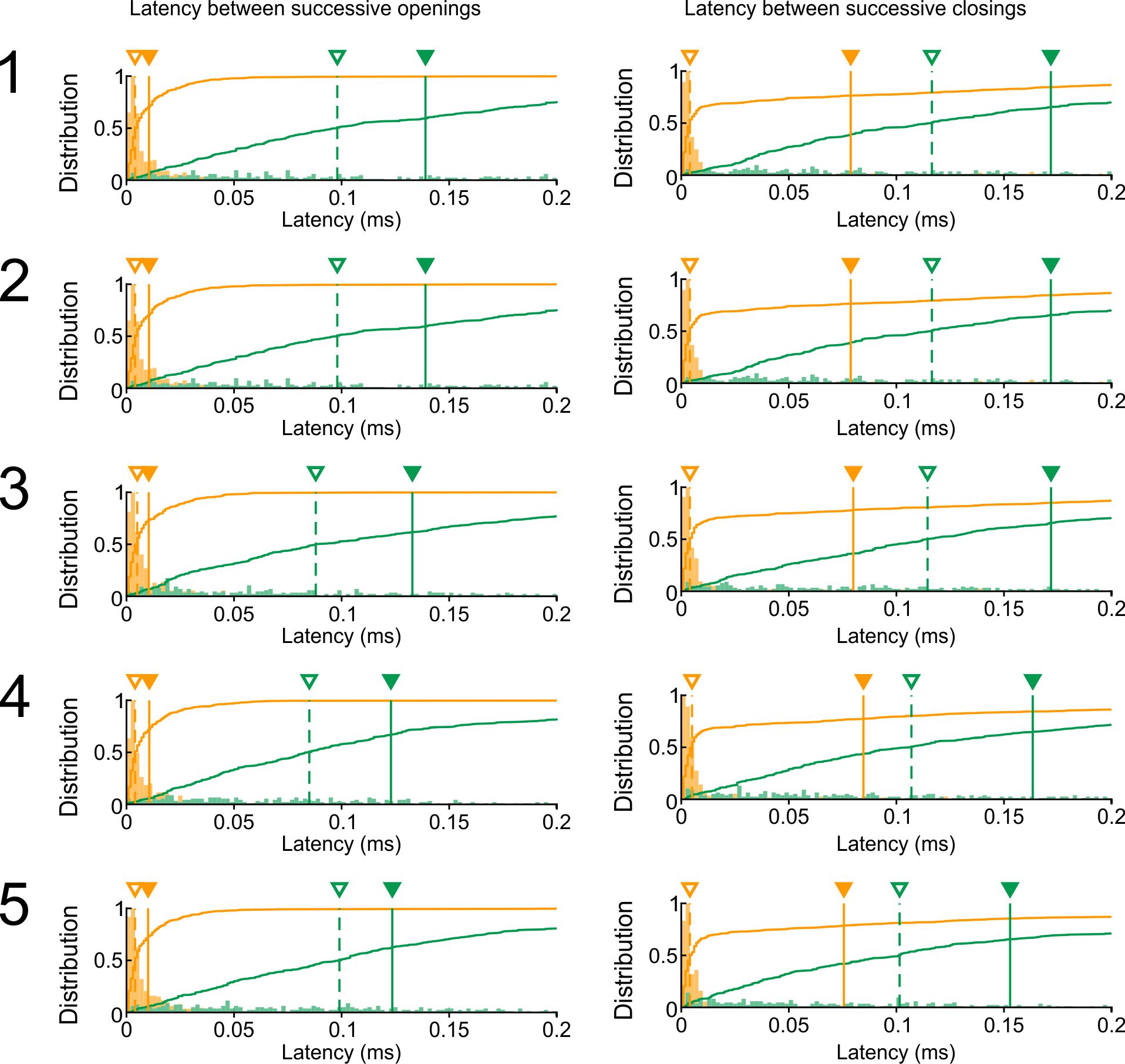


**Supplementary Figure S7.** Histograms of the latencies between successive openings (*left*) and of the latencies between successive closings (*right*) for 5 different realizations of the simulation with the wild-type Clancy-Rudy model pair without interaction (green) and with Interaction II (orange). Same layout as in Figure 9C of the main article. The solid curves represent cumulated histograms. Filled triangles and solid vertical lines indicate means; open triangles and dashed vertical lines indicate median values. Although not exactly identical due the stochastic nature of the simulations, the histograms all have a similar aspect.


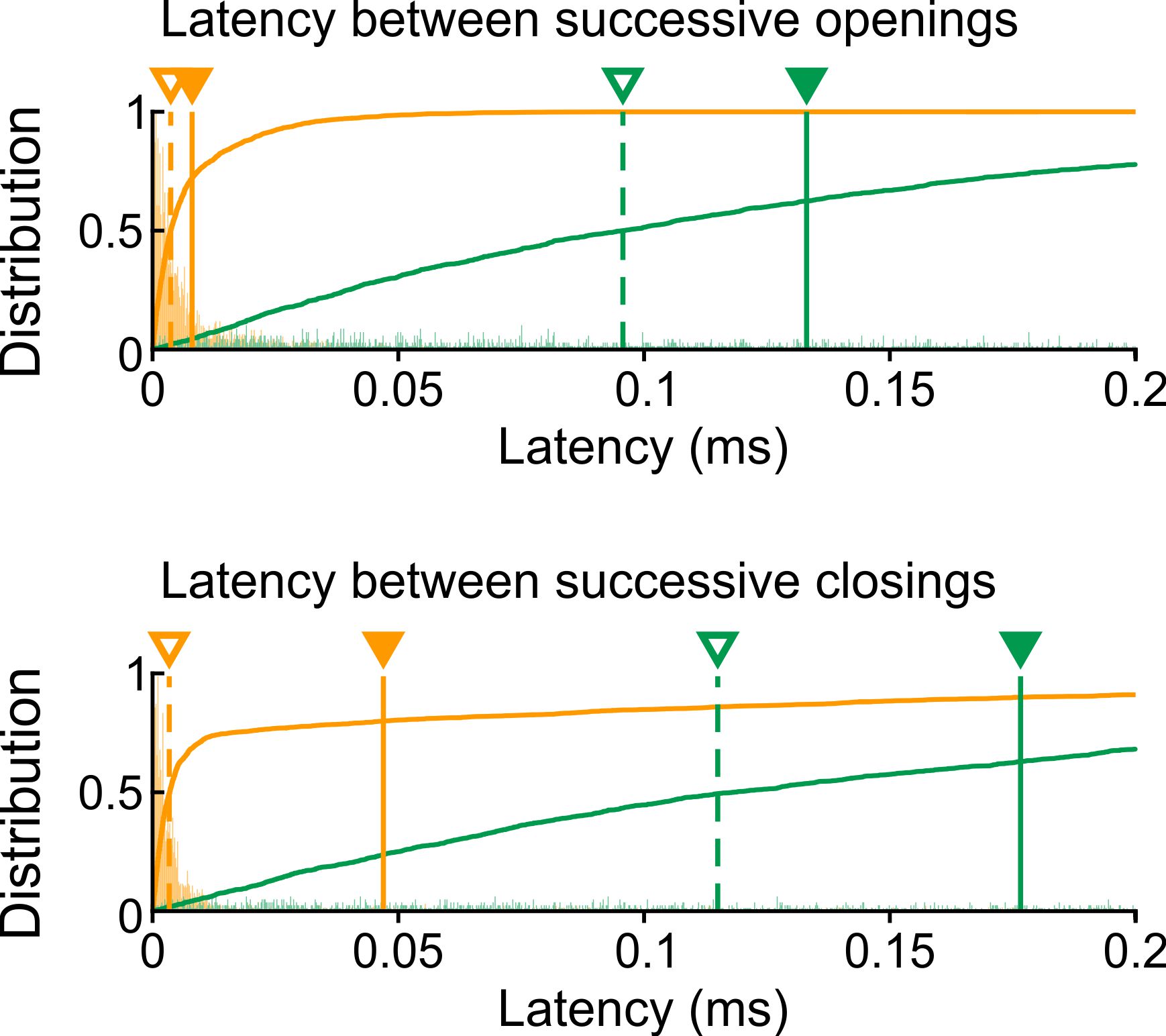


**Supplementary Figure S8.** Histograms of the latencies between successive openings (*top*) and of the latencies between successive closings (*right*) for the simulated wild-type Clancy-Rudy model pair without interaction (green) and with Interaction II (orange), constructed using a 10x shorter time step Δt of 0.0001 ms and using narrower bins. For this simulation, the number of sweeps *n* was increased 10x to 10000. Same layout as in Figure 9C of the main article and in Supplementary Figure S7. The figure shows that the histograms do not exhibit peaks or modes but are monotonically decreasing.
